# Synthesis and Characterization of Carbon-Based Heterogeneous Catalysts for Energy Release of Molecular Solar Thermal Energy Storage Materials

**DOI:** 10.1021/acsami.3c16855

**Published:** 2024-02-01

**Authors:** Lucien Magson, Helen Hölzel, Adil S. Aslam, Stefan Henninger, Gunther Munz, Kasper Moth-Poulsen, Markus Knaebbeler-Buss, Ignacio Funes-Ardoiz, Diego Sampedro

**Affiliations:** † Instituto de Investigación en Química de la Universidad de La Rioja (IQUR), C/Madre de Dios 53, Logroño 26004, La Rioja; ‡ Department of Chemistry and Chemical Engineering, Chalmers University of Technology, Kemivagen 4, Gothenburg 412 96, Sweden; § Department of Chemical Engineering, Universitat Politècnica de Catalunya, EEBE, Eduard Maristany 10-14, Barcelona 08019, Spain; ∥ Heating and Cooling Technologies, Fraunhofer Institute for Solar Energy Systems (ISE), Heidenhofstr. 2, Freiburg 79110, Germany; ⊥ Catalan Institution for Research & Advanced Studies, ICREA, Pg. Llúıs Companys 23, Barcelona 08010, Spain; # Institute of Materials Science of Barcelona, ICMAB-CSIC, Bellaterra, Barcelona 08193, Spain; ¶ Hydrogen Technologies and Electrical Energy Storage, Fraunhofer Institute for Solar Energy Systems (ISE), Heidenhofstr. 2, Freiburg 79110, Germany

**Keywords:** heterogeneous catalysts, abundant metals, energy storage, MOST systems, isomerization

## Abstract

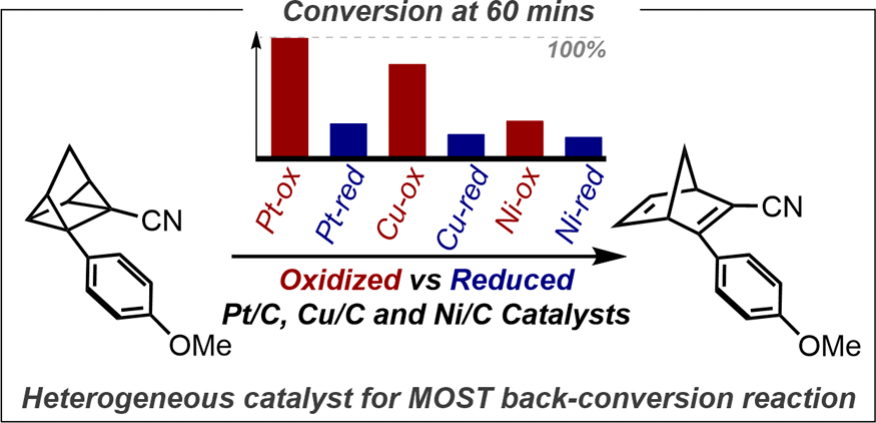

Molecular solar thermal energy storage (MOST) systems are rapidly becoming a feasible alternative to energy storage and net-zero carbon emission heating. MOST systems involve a single photoisomerization pair that incorporates light absorption, storage, and heat release processes in one recurring cycle. Despite significant recent advancements in the field, the catalytic back-reaction from MOST systems remains relatively unexplored. A wide range of applications is possible, contingent on the energy densities of the specific photoisomers. Here, we report platinum-, copper-, and nickel-based heterogeneous catalysts screened in batch conditions for the back-conversion reaction on the cyano-3-(4-methoxyphenyl)-norbornadiene/quadricyclane pair. Catalyst reactivities are investigated using structural characterization, imaging techniques, and spectroscopic analysis. Finally, the thermal stability is also explored for our best-performing catalysts.

## Introduction

According to the World Energy Outlook 2022 report, the combination of the COVID pandemic and the current energy crisis means around 70 million people who recently gained access to electricity will lose the ability to afford it.^1^ The recent energy crisis represents a monumental turning point toward cleaner energy sources to combat the ever-growing visible impacts of climate change.

Solar energy represents an abundant source for clean energy production where annually the earth’s surface is struck by 79,000 Terawatts of incoming solar radiation. On a typical summer day, an equivalent of 12 h of solar radiation would supply approximately double the global annual energy consumption.^2^ A key obstacle in the application of solar energy systems is the intermittent character of solar radiation supply, which varies temporally and geographically. Solar radiation which reaches the earth’s surface is called direct beam solar radiation. Sunlight penetration through the atmosphere can be absorbed, scattered, and reflected by air molecules, water vapor, clouds, pollutants, dust, and forest fires causing strong mismatches in load leveling.^3^ Consequently, it is of paramount importance to expedite the development of novel solar energy storage technologies to provide sufficient and reliable energy.

Many industrial reactions continue to generate chemical fuels using noble metal catalysts as the active phase such as Pd, Pt, Ru, and Rh due to their high activity, selectivity, and stability under demanding reaction conditions.^4−6^ For example, in the hydrogen evolution reaction (HER) in proton exchange membrane (PEM) fuel cells, Pt has the highest activity relative to that of first-row transition metals. In HER reactions, an optimal intermediary strength of catalyst-substrate interaction occurs with Pt, a key reason for its high catalytic activity.^7^ Recently, catalytic strategies have involved reducing the concentration of noble metals and maximizing the catalytic surface area exposed to reactants via hollow nanoparticles,^8^ single-atoms,^9^ fully exposed few-atom clusters,^10^ metal organic frameworks,^11^ and alloys integrating abundant metals.^12^ These advancements improve the economic feasibility of the catalytic process and can become a sustainable manner to scale up these reactions.

**Figure 1:**
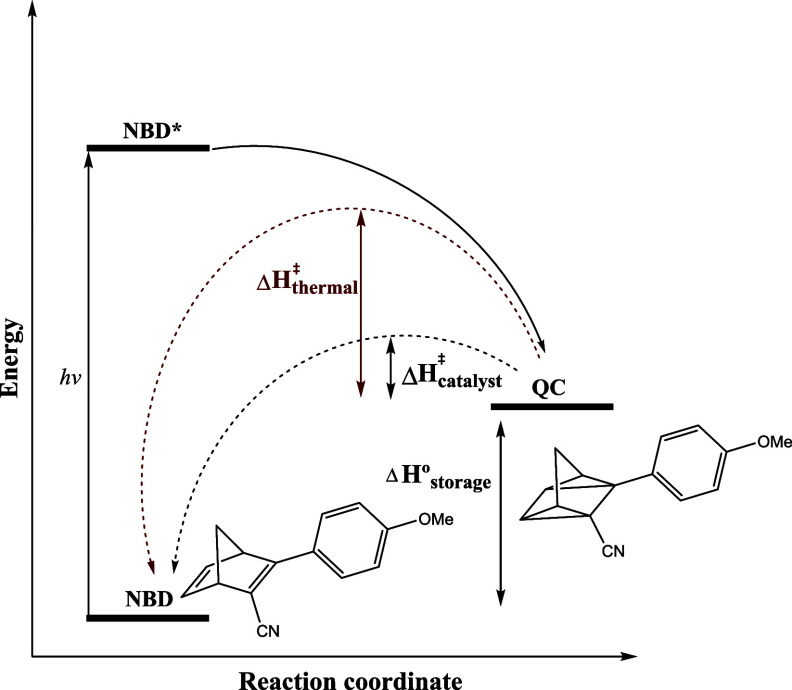
Schematic energy diagram of the norbornadiene-quadricyclane photo isomer system.^14^

A scarcely investigated area is the use of molecular solar thermal energy storage systems (MOST) otherwise known as “solar thermal fuels”, which are composed of pairs of isomers that act like rechargeable batteries. The parent isomer undergoes light absorption, transforming to a metastable isomer. The metastable isomer can have varying half-lives between hours and years and energy densities of up to 0.4 MJ kg^–1^.^13^ The stored energy can be released in the form of heat, and this reaction can be triggered either catalytically, electrochemically, optically, or thermally, returning to its parent isomer (Figure 1).^14−18^ Unlike the previously mentioned solar-driven to chemical fuel processes, the reaction media are not acidic, and no intake or emission from external sources (O_2_, H_2_O, or CO) is necessary. Hence, charging and energy extraction reactions can all be completed within a closed cycle.

Among MOST systems, the norbornadiene and quadricyclane couple remains the most investigated system, yet the catalytic back reaction to release the stored energy is relatively unexplored. Homogeneous catalysis involves a catalyst and a reaction mixture within the same phase. These catalysts exhibit several advantages, including a high degree of interaction, good selectivity, and absence of mass-transport limitations. Currently, cobalt phthalocyanine complexes have been a viable way of releasing the metastable isomer’s stored energy showing moderate activity in converting quadricyclane to norbornadiene without side products.^14,19^ Despite nonexistent phase boundaries in homogeneous catalysis, laborious procedures including distillation and extraction are required to separate the catalyst from the reaction mixture, making them economically unviable.

In heterogeneous catalysts, the active sites are immobilized onto a solid support, in which reaction mixtures can easily be separated by filtration, or in a fixed bed format, no filtration is necessary. Moreover, many solid supports are porous to maximize their high surface-area-to-volume ratio, increasing the available concentration of active sites. In addition, when the reaction mixture moves to higher concentration, the likelihood of surface saturation drastically reduces. Recently, Moth-Poulsen et al. physiosorbed cobalt phthalocyanine complexes onto activated carbon to generate a high reaction surface area, ultimately leading to an increase in reaction rate; 69 times higher than for untreated CoPc.^14^

Heterogenization is not the sole requirement when scaling up toward industrial levels, and eliminating our use of precious, expensive metals is primordial to finding a truly renewable and cost-effective energy solution within our current market. Thus, two first-row transition metals were selected and compared against a precious metal catalyst. Here, we report three reduced platinum, copper, and nickel heterogeneous catalysts and three oxidized counterparts screened for the back-reaction of the same push–pull-substituted norbornadiene-quadricyclane photoisomer. Structure–reactivity relationships of these six heterogeneous catalysts were characterized by using XRD, SEM, nitrogen physisorption, DVS, DSC, and DRIFTS.

## Experimental Methods

### Synthesis of Oxidized and Reduced Catalysts

The Pt-Ox, Cu-Ox, and Ni-Ox supported (nominal 5.0 ± 0.5.-wt % metal) activated carbon catalyst was synthesized by the conventional impregnation method (i.e., incipient wetness method). A Norit SX Plus activated carbon was initially washed in deionized water and dried overnight at 120 °C in a Nabertherm muffle oven. Here, 95.0 mg of activated carbon was weighed in, and a 5 wt % of metal was added for each catalyst. 13.3 mg of dihydrogen hexachloroplatinate (IV) hexahydrate precursor, 19.0 mg of copper(II) nitrate trihydrate, and 28.0 mg of nickel(II) acetate tetrahydrate were used. The pore volume of the Norit SX Plus activated carbon is 605 μL/g; thus, to fill the exact pore volume, each metal precursor was dissolved in 57.5 μL of ethanol (apart from nickel acetate tetrahydrate which was dissolved in deionized water), and sonication was used to aid dissolution. The precursor solution was added dropwise using a syringe pump at a rate of 10 μL/min to the carbon support. The sample was mixed thoroughly prior to being placed in the muffle oven. First, a ramp in temperature was applied from 25 to 120 °C at 60 °C/h, the temperature was held at 120 °C for 2 h, next a ramp in temperature from 120 to 300 °C at 60 °C/h was done, and finally, the samples were calcined at 300 °C for 12 h. The sample was cooled to room temperature and stored in an airtight vial prior to use.

The Pt-Red, Cu-Red, and Ni-Red supported activated carbon catalyst was synthesized also using the incipient wetness method. All details of the impregnation method remained the same as described for the oxidized versions. The calcination procedure was performed in a tubular reactor, where the samples were placed in a ceramic tray. First, a ramp up in temperature was applied from 25 to 120 °C at 60 °C/h, and the temperature was held at 120 °C for 2 h.

The Pt-Red and Ni-Red samples were calcined at 300 °C under nitrogen flow (100 mL/min) for 12 h. Next, a hydrogen flow (100 mL/min) was applied for 2 h, maintaining a temperature of 300 °C to do the reduction. The samples were cooled to room temperature in a nitrogen flow of 100 mL/min and stored in an airtight vial prior to use.

The Cu-Red sample was calcined at 300 °C under argon flow (100 mL/min) for 12 h. Next, a hydrogen flow (100 mL/min) was applied for 2 h maintaining a temperature of 300 °C. The samples were cooled to room temperature in a nitrogen flow of 100 mL/min and stored in an airtight vial prior to use.

### Characterization

Powder X-ray diffraction patterns were recorded using a Rigaku Diffractometer using Cu Kα radiation (λ = 1.54 Å). The structure and morphology were determined by SEM performed on a Zeiss Gemini 360 instrument. The specific surface area calculated using the Brunauer–Emmett–Teller method, the pore volume, and the pore size distribution were determined by nitrogen adsorption–desorption isotherm measurements at 77 K on an Anton Paar QuadraSorb instrument. Diffuse reflectance Fourier transform infrared (DRIFTS) spectra were recorded by using a PerkinElmer Lambda Spectrum Two FTIR instrument. Metal loadings were measured by using a PinAAcle 500 Flame Atomic Absorption Spectrometer instrument. Water uptake was measured using an Anton Paar QuantaTec Vstar instrument. Evaluation of thermal stabilities was done through differential scanning calorimetry (DSC) measurements using a Mettler-Toledo (DSC2A-00312) instrument. NMR characterization of the norbornadiene and quadricyclane pair was accomplished by using a Bruker 400 MHz spectrometer instrument.

### Catalytic Activity Measurements

Catalytic reactions were performed using the following protocol. A 4.48 × 10^–3^ M 10 mL solution (1 mg/mL) of NBD was prepared in toluene. The NBD solution was photoirradiated using a UVA (315–400 nm with a maximum at 350 nm) light source for 90 min. The catalytic reaction is started once less than 2% norbornadiene is present in the solution. The catalysts were screened using a 10% weight ratio of catalyst (1 mg) and stirred at a rate of 500 rpm in the 1 mg/mL photoirradiated 10 mL norbornadiene solution. The catalytic reaction was monitored at specific time intervals. A 1 in 100 dilution in toluene and catalyst separation from the reaction mixture using 0.22 μm filters were done prior to quantification via UV–vis measurements. The isomerization reaction is quantified by the formation of norbornadiene using a Shimadzu UV–vis spectrometer. The absorbance value at 340 nm is taken as no overlap occurs with the absorption band of quadricyclane and has a high linearity in the concentration range (see in Figure S34).

## Results and Discussion

### Synthesis and Characterization

Two classes of heterogeneous catalysts were synthesized using the incipient wetness impregnation method,^20^ where all catalysts are tethered to the same commercially available Norit SX Plus powdered activated carbon support. The three metal precursors chosen were dihydrogen hexachloroplatinate(IV) hexahydrate, copper(II) nitrate trihydrate, and nickel(II) acetate tetrahydrate, where a 5% metal loading by weight was used for each catalyst. The key difference between the two classes of catalysts synthesized is the calcination procedure. Three catalysts of platinum, copper, and nickel were calcined in a tubular reactor with a reducing hydrogen flow, while the other three platinum, copper, and nickel catalysts were calcined using a muffle furnace in an oxidizing atmosphere. The same calcination temperature of 300 °C was used for all six catalysts (see Synthesis in the Supporting Information). By changing the calcination atmosphere, the metals will have different oxidation states, the metal–support interactions will vary, and the location, size distinctions, and distribution of metal crystallites on the support will fluctuate, all causing differences in the rates of reaction of the quadricyclane to norbornadiene transformation.

**Figure 2:**
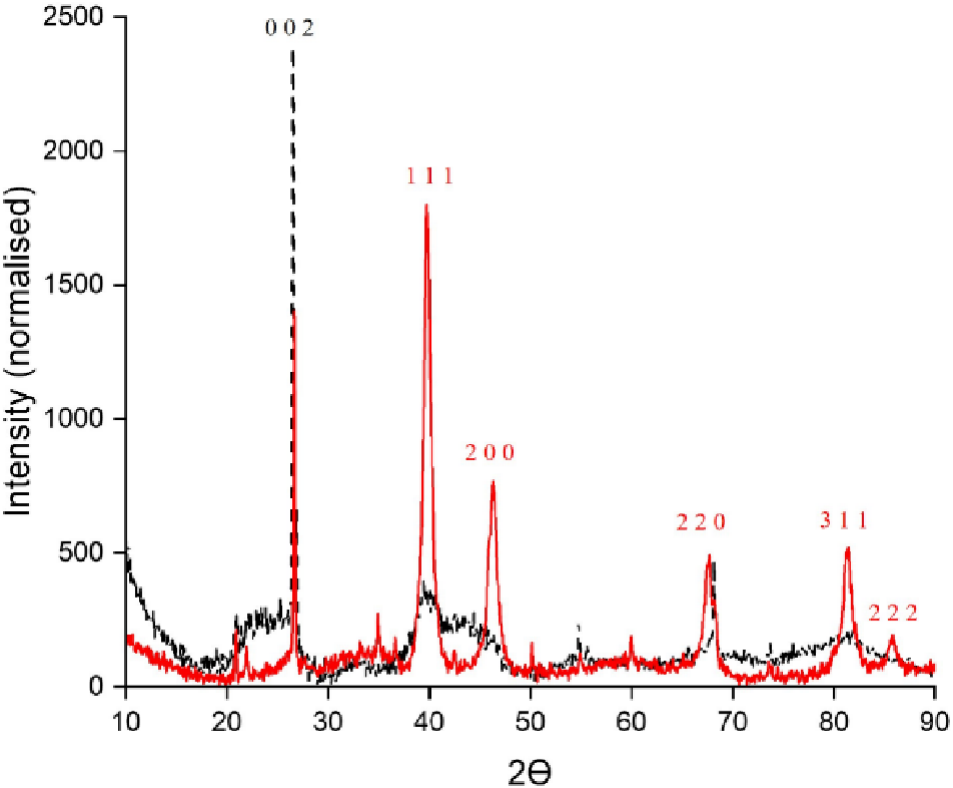
Pt-Red (black) and Pt-Ox (red) with the diffraction planes shown above.

Powder X-ray diffraction (PXRD) was recorded for the reduced and oxidized catalysts to identify changes in phase composition, the nature of the arranged constituent particles, and crystallite sizes. PXRD results for the two platinum catalysts (Figure 2) demonstrate that calcining in a controlled reducing hydrogen atmosphere produces a disordered amorphous catalyst. A broad poorly resolved major (111) diffraction peak with an uneven baseline is observed, suggesting a wide size distribution of platinum particles. In contrast, in an oxidizing atmosphere, oxygen plays a crucial role in organizing platinum crystallites into a well-ordered crystal lattice. Qualitative phase identification confirms platinum takes an *Fm*3̅*m* (225) space group whereas at high oxygen pressures and high temperatures, a β-PtO_2_ orthorhombic structure is created.^21^ Additionally, the peaks exhibit sharp tips without tails, implying that a very uniform size distribution is present.

**Figure 3:**
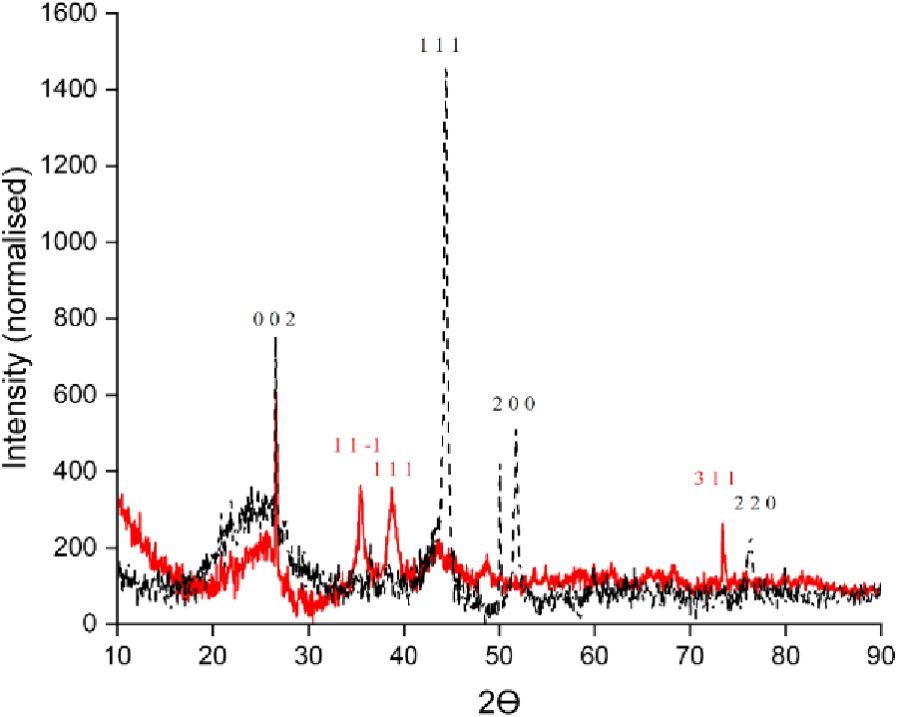
Cu-Red (black) and Cu-Ox (red) with the diffraction planes shown above.

In the case of copper (Figure 3), both catalysts produced a crystalline arrangement. For Cu-Red, an *Fm*3̅*m* (225) space group, identical to Pt-Red appears although a more common fluorite structure is produced. For Cu-Ox, two equally intense peaks are displayed belonging to a tenorite (CuO) structure.^22^ The tenorite structure has a monoclinic symmetry taking the *C* 2/*c* space group, where the Cu and O atoms are in a prismatic polyhedral structure.

**Figure 4:**
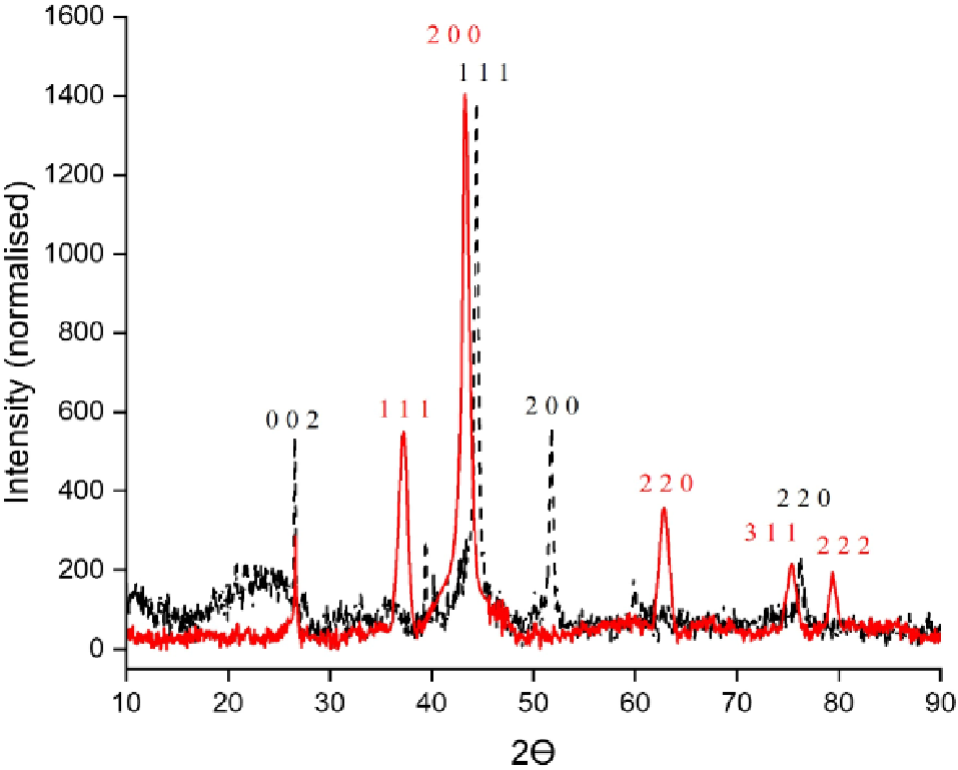
Ni-Red (black) and Ni-Ox (red) with the diffraction planes shown above.

Similarly, for nickel, both reduced and oxidized catalysts produce a crystalline arrangement that takes a *Fm*3̅*m* (225) space group (Figure 4).^23^ In all the XRD patterns, a moderately sharp diffraction peak for carbon appears at approximately 26.5° indicating a high degree of graphitization.^24^

X-ray diffraction is a convenient method for determining the mean size of the crystallites within the bulk material. The Debye–Scherrer equation accounts for broadening predominantly due to crystallite size, in which broader peaks indicate smaller crystallite sizes (see Figure S1). Consequently, in Table 1, the crystallite size is cross-referenced with the metal loading by weight per catalyst to be able to compare nanoparticle growth and distribution effects.

**Table 1:** Nanoparticle Size Was Calculated from the Most Intense Diffraction Peaks for Three Reduced and Three Oxidized Catalysts

catalyst	crystallite size (nm)	metal loading (%)^a^
Pt-Red	b	5.8
Pt-Ox	20	8.1
Cu-Red	47	6.6
Cu-Ox	33	5.9
Ni-Red	46	8.1
Ni-Ox	22	6.8

a Metal loading measured using atomic absorption spectroscopy (AAS).

b Not applicable using this method.

All of the oxidized catalysts exhibit smaller crystallite sizes relative to their reduced counterparts. Considering that the metal loading varies minimally across all six catalysts, the crystallite growth is lower for oxidized catalysts, implying a higher surface area to volume ratio and a greater dispersion of active sites across the activated carbon. Despite the increased surface free energy of the oxidized catalysts, these crystallites become more prone to sintering through Ostwald ripening or via the formation of agglomerates and adlayers.^25^

**Figure 5:**
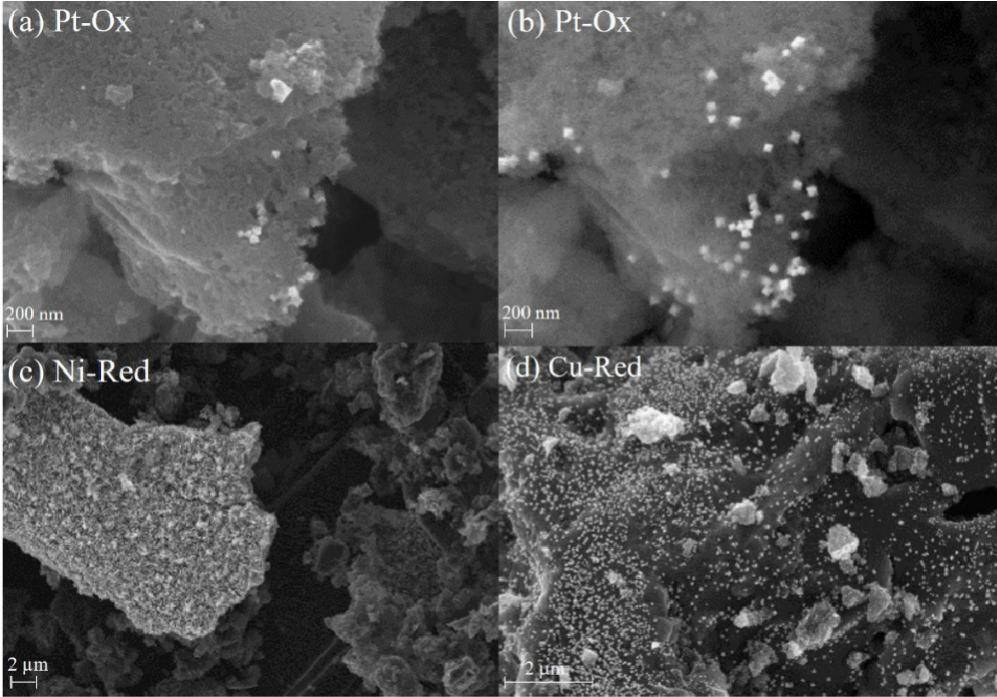
High-resolution SEM images of the Pt-Ox catalyst using an in-Lens detector (a) and an SE2 detector (b), Ni-Red catalyst (c), and Cu-Red catalyst (d) images using an in-Lens detector.

High-resolution SEM images were taken to assess the morphology of Pt-Ox, Cu-Red, and Ni-Red catalysts, including the size, shape, distribution, and location of metal crystallites across the activated carbon (Figure 5). The top two images are of the Pt-Ox catalyst which differ only via the detector used; an in-Lens detector (a) is used which has a lower electron beam penetration depth than the secondary electron detector (b). A partial embedding of platinum crystallites within the porous network occurs. Taking into consideration that catalytic reactions involve a solid–liquid interface, catalyst leaching from its support may be reduced. Moreover, Pt-Ox nanoparticles are octahedral shaped with well-defined facets and sharp edges, enhancing their activity. Upon further inspection, a uniform and homogeneous distribution of platinum crystallites is found to be located across both the “basal” and the “edge” planes of the graphitic sheets of activated carbon (see Figure S11).

For both Ni- and Cu-Red catalysts, crystallites are located on the basal planes of the activated carbon. Here, minimal embedding of metal crystallites within the porous regions occurs with the catalysts taking on an “eggshell” structure, shown further in Figures S6 and S7. In the case of Ni-Red (c), a very uniform and wide distribution of nanorod-shaped particles is present. While for Cu-Red (d), a very uniform and homogeneous distribution of spherically shaped copper particles appears.

Diffuse reflectance infrared spectroscopy (DRIFTS) was carried out on all six catalysts (see Figures S12 and S13). All the reduced catalysts display identical absorbance peaks as the pure activated carbon support, indicating the functional groups on the activated carbon remain intact after impregnation. Corresponding with the SEM images, a π-cation noncovalent interaction is present between metal and electron-rich groups like lactones or carboxylic anhydrides.^26^ In the Ni-Ox catalyst, multiple peaks appear at 920 cm^–1^ and 1300 cm^–1^ belonging to C–O stretching in lactones and carboxylic anhydrides. Plus, an extra C=C aromatic stretching peak at 1586 cm^–1^ and a peak at 1697 cm^–1^ confirm the presence of a carbonyl species like quinone and/or chromene group.^27,28^ In the case of the Pt-Ox catalyst, an extra C=O stretching at 1790 cm^–1^ from a lactone or carboxylic anhydride group is present. Finally, in the Cu-Ox catalyst, no additional observable peaks are visible in the spectra, displaying similar distinguishing features as the carbon support.

The textural properties of the six heterogeneous catalysts were examined by physisorption using N_2_ as a probe molecule at 77 K. Activity relationships were evaluated by analyzing the surface areas, pore volumes, and pore sizes of these heterogeneous catalysts (Table 2).

**Table 2:** BET Data of Pt, Cu, and Ni on Activated Carbon Catalysts wherein the Pore Volume and Pore Size Were Calculated Using a Slit/Cylindrical/Sphere Pore, QSDFT Adsorption Branch Model, and DR Method, Respectively

catalyst	BET surface area (m^2^/g)	pore volume (cc/g)	pore size (nm)
Pt-Ox	1070	0.54	9.5
Cu-Ox	1090	0.58	9.7
Ni-Ox	1020	0.51	9.2
Pt-Red	1030	0.62	8.9
Cu-Red	889	0.65	8.8
Ni-Red	854	0.66	9.1
Norit SX Plus	989	0.58	8.9

Complementary to SEM images, a considerably lower pore volume is recorded for both Pt-Ox and Cu-Ox catalysts relative to their reduced opposite number, agreeing with a greater uptake of metal ions into mesopores. Using an oxidizing atmosphere, a higher percentage of oxygenated groups is present on the carbon surface, underlining the greater ability for the diffusion of metal species across the carbon surface. In addition, a higher surface area and pore size arise for the oxidized catalysts relative to their counterpart, suggesting a greater degree of pore opening.

The nitrogen isotherm in Figures S17–S23 exhibits a type II isotherm which signifies mesoporous sizes extending into the macroporous range. The hysteresis between adsorption and desorption isotherms is classed as H3 indicating a slitlike pore structure often found in loose assemblies of platelike particles like activated carbon. The use of activated carbon with a broad mesoporosity enables a high surface area for an even distribution of active metal sites.^26^ Moreover, having a high percentage of mesopores mixed with macropores allows for fast transfer kinetics between reactants and active sites.

**Figure 6:**
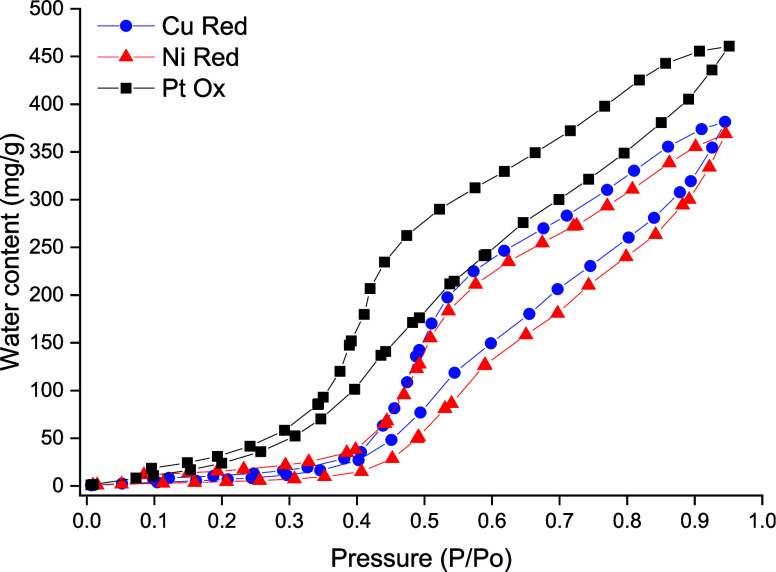
Water vapor sorption isotherms of Pt-Ox, Cu-Red, and Ni-Red catalysts at varying pressures.

Water vapor sorption experiments were performed to determine the quantity of water adsorbed by the Pt-Ox, Cu-Red, and Ni-Red catalysts at varying pressures (Figure 6). The norbornadiene-quadricyclane pairing proposed for this MOST device is quite hydrophobic; thus, for optimal mass-transfer kinetics, the heterogeneous catalysts need to be hydrophobic too.

A very positive outcome is that the total uptake of water is low over the tested relative pressure range. For Pt-Ox, a minor increase in the uptake of water occurs, indicating the increase in the percentage of oxygen-containing functional groups on the activated carbon support. However, the adsorption isotherm steadily increases toward the higher-pressure ranges, signifying that the uptake of water is diffusion-limited. Thus, it is probable that both surface and bulk adsorption processes occur on these catalysts.

**Figure 7:**
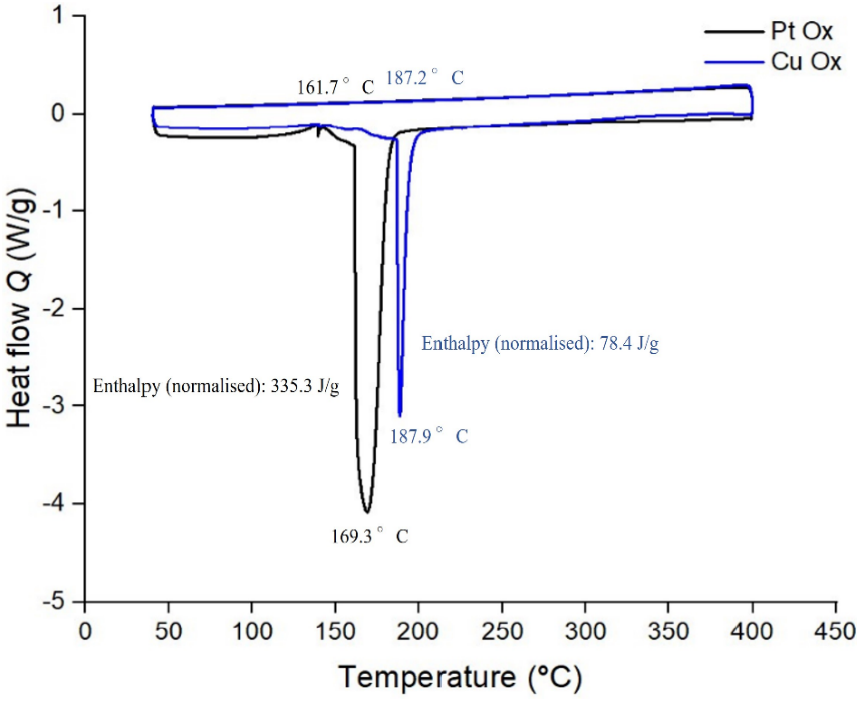
DSC heating and cooling cycles for Pt-Ox (black) and Cu-Ox (blue) catalysts at a ramp rate of 3 K/min.

Differential scanning calorimetry measurements were performed for the Pt-Ox and Cu-Ox catalysts to study their thermal stability and polymorphic behavior at elevated temperatures (Figure 7). In both instances, we have a solid–solid polymorphic transition whereby after performing a heating cycle, the catalyst remains in powder form. After a freezing cycle was made, no reversible transition process from the inactive β form to the active α form materializes. Further confirmation of a monotropic transition was done, wherein after a second heating cycle of the same sample, no phase change peak is seen (see Figures S26–S31).

For the Pt-Ox catalyst, a monotropic solid-to-solid transition that is initially concave in shape suggests minor impurities of a different phase are present. The transition to the inactive polymorph is recorded at a peak maximum of 169 °C. Interestingly, for the Cu-Ox catalyst, the monotropic solid-to-solid transition produces a much sharper peak at higher temperatures, which highlights a higher degree of crystallinity with a more homogeneous size distribution of crystallites and high sample purity. As a straight line is observed, the onset of the transition peak is taken at 187.2 °C. The thermal stability of the active polymorphs is in the exact temperature range required for steam generation; an application previously proposed for the MOST system.^14^

### Catalytic Performance on the QC-NBD Back-Conversion

Considering the commercially available activated carbon support exhibits high attrition resistance (reduced catalyst losses via fines generation) and good suspension characteristics (resistance to mass transfer effects), batch reactions of powdered catalysts were accomplished, creating slurries with the liquid phase reactants. Catalytic batch reactions were quantified by the formation of norbornadiene and monitored over specific time intervals.

**Figure 8:**
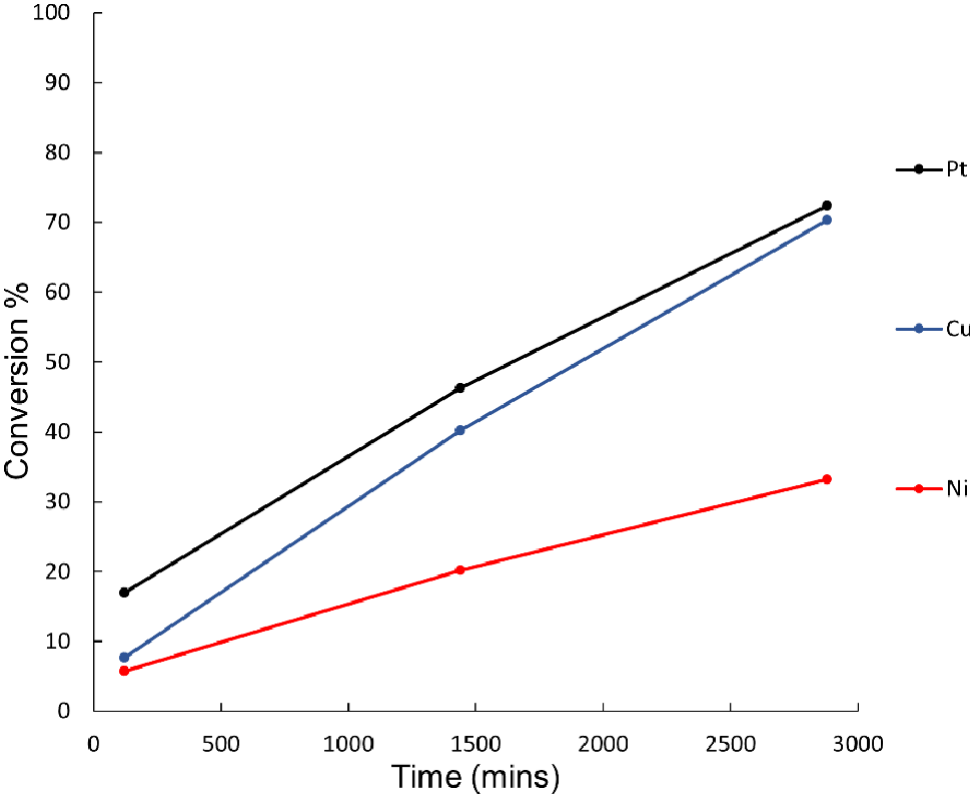
Catalytic back-conversion of Pt-Red, Cu-Red, and Ni-Red catalysts, when using a 10% weight loading of catalyst relative to a 1 mg/mL photoirradiated solution of norbornadiene dissolved in toluene.

An initial screening of activity for Pt-Red, Cu-Red, and Ni-Red catalysts calcined in a reducing hydrogen flow was performed for the quadricyclane to norbornadiene isomerization reaction (Figure 8).

**Figure 9:**
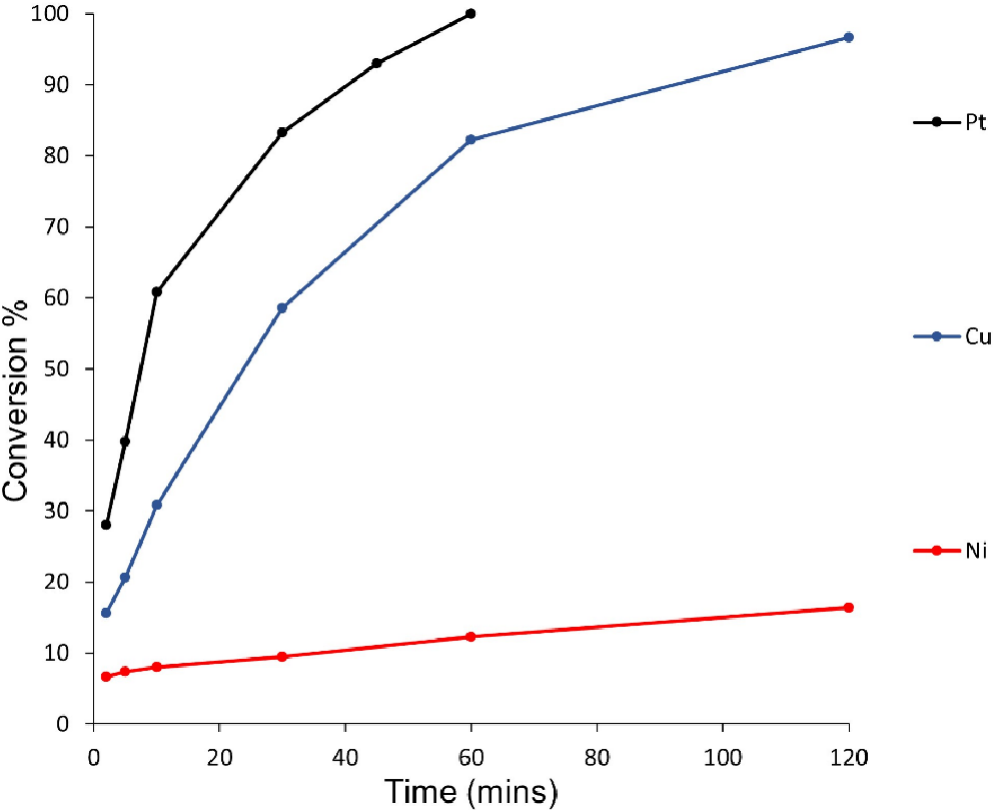
Catalytic back-conversion of Pt-Ox, Cu-Ox, and Ni-Ox catalysts, when using a 10% weight loading of catalyst relative to a 1 mg/mL photoirradiated solution of norbornadiene dissolved in toluene.

All of the reduced catalysts demonstrate very low activities for the isomerization reaction. A second screening for Pt-Ox, Cu-Ox, and Ni-Ox catalysts calcined in an oxidizing atmosphere was performed showing improved activities aside from Ni-Ox (Figure 9).

In heterogeneous catalysis, for batch reactions, the reaction rate is dependent on microkinetic parameters, including the concentration of the reactants, the temperature, and the catalyst. In this set of reactions, the reaction rate can be expressed relative to the specific surface area *S* of the catalyst (m^2^ kg^–1^) (see Table S3 in the Supporting Information).^29^ All three oxidized catalysts demonstrate significantly greater activities for this back-reaction, increasing 100-fold with respect to the reduced counterparts.

Considering the large uptake of metal species into the mesoporous channels of activated carbon, the oxidized catalysts exhibit a higher activity per unit mass of metal compared to the reduced catalysts. Moving to metal oxides strongly enhances the metal’s covalent character from their formal oxidation states.^30^ For example, in the most active catalyst, platinum oxidation states strongly deviate from Pt^2+^ and Pt^4+^.^31,32^ These fractional oxidation states result in partial occupation of the highest d orbital, meaning platinum has a high electron affinity. Similarly, PXRD of the copper catalyst confirms a tenorite structure whereby a + 2-oxidation state is present for copper (3d^9^); this means the oxide has a much higher electron affinity than copper (3d^10^ 4s^1^). With the added covalent character of mixed valent oxide systems, stronger interactions of the platinum are allowed with the strained carbon atom on the quadricyclane core structure.

Batch experiments were conducted for Pt-Ox and Cu-Ox catalysts calcined at temperatures ranging from 200 to 500 °C. The optimal calcination temperature was verified at 300 °C (see Figures S36 and S37). Additionally, batch experiments were performed on water-soaked and vacuum-dried Cu-Ox and Cu-Red catalysts to determine water’s impact on catalyst performance (see Figure S35) The effect is substantial where conversion is *circa* four times slower for the Cu-Ox catalyst and twice as slow for the reduced catalyst after 2 h. Upon increasing the moisture content, aside from physical changes such as swelling and partial disintegration of the activated carbon, small deviations in the catalyst structure can occur including the materialization of oxidized carbon species,^33^ surface hydroxyls,^33^ adsorbed water,^34^ and undercoordinated oxygen sites,^35^ which can disrupt the crystalline arrangement of copper nanoparticles. Considering both catalytic back-reactions over long time periods are notably weak, it is likely that competition occurs between solvent and reactant molecules for active sites. Thus, maintaining a closed catalytic environment is necessary, as the quadricyclane to norbornadiene reaction is drastically hindered in the presence of hydrogen bonding solvents like water.

In this article, not only do expensive platinum metals function very fast in initiating the catalytic back-reaction but, fortunately, more abundant, cheaper metals like copper display high activity too. Considering the mild reaction conditions and due to the good recyclability of copper, larger-scale implementation of these solid-supported catalysts in the form of a fixed-bed reactor would be economically feasible.

## Conclusion

As a class of catalysts, porous supported metal catalysts calcined in an oxidizing atmosphere display very high catalytic rates of reaction, in which, to the best of our knowledge, the Pt-Ox catalyst produces the highest rate of reaction for the transformation of quadricyclane to norbornadiene to date. Encouragingly, abundant, cheaper metals such as copper produce very similar reaction rates and are thermally stable at elevated temperatures. Furthermore, Norit SX Plus is a porous activated carbon with graphitic domains that contains a high density of acidic groups available to interact with various metals. Thus, it proves to be a very good catalytic support, demonstrating minimal mass transport limitations. The combination of a high energy density norbornadiene-quadricyclane MOST photoisomer of 0.4 MJ kg^–1^ that can store solar energy for long times and using a copper-based catalyst which attains 82% conversion in 1 h would directly address our over-reliance on precious metals in this green energy transition.

## Supporting Information Available

Additional experimental details including synthesis, characterization, and kinetic measurements (PDF)

## ABBREVIATIONS

MOST: molecular solar thermal energy storage system
NBD: norbornadiene
QC: quadricyclane
PXRD: powder X-ray diffraction
SEM: scanning electron microscopy
BET: Brunauer–Emmett–Teller
DVS: dynamic vapor sorption
DSC: differential scanning calorimetry
DRIFTS: diffuse reflectance Fourier transform infrared spectroscopy
AAS: atomic absorption spectroscopy
