# Supplementary material for: Synthesis and Characterization of Carbon-Based Heterogeneous Catalysts for Energy Release of Molecular Solar Thermal Energy Storage Materials
Source: ACS Appl Mater Interfaces. 2024 Feb 1;16(6):7211–8. doi: 10.1021/acsami.3c16855 (PMC10875640; doi:10.1021/acsami.3c16855)
Supplement: Supplementary file 1 — am3c16855_si_001.pdf [file am3c16855_si_001.pdf]

<u>**Supporting Information**</u>

Synthesis and Characterization of Carbon-based Heterogeneous Catalysts for Energy Release of Molecular Solar Thermal Energy Storage Materials

Lucien Magson, Helen Hölzel, Adil S. Aslam, Stefan Henninger, Gunther Munz, Kasper Moth-Poulsen, Markus Knaebbeler-Buss,* Ignacio Funes-Ardoiz* and Diego Sampedro*

L. Magson, Dr. I. Funes-Ardoiz and Prof. Dr. D. Sampedro
Centro de Investigación en Síntesis Química
Universidad de La Rioja
C/Madre de Dios 53, 26004 Logroño, La Rioja
Ignacio.funesa@unirioja.es; Diego.sampedro@unirioja.es

Dr. H Hölzel, Dr. A. S. Aslam and Prof. K. Moth-Poulsen
Department of Chemistry and Chemical Engineering, Chalmers University of Technology, Kemivagen 4, Gothenburg 412 96, Sweden.
Dr. H Hölzel, and Prof. K. Moth-Poulsen
Department of Chemical Engineering, Universitat Politècnica de Catalunya, EEBE, Eduard Maristany 10-14, 08019 Barcelona, Spain.
Prof. K. Moth-Poulsen
Catalan Institution for Research & Advanced Studies, ICREA, Pg. Llu´ıs Companys 23, Barcelona, Spain.
Prof. K. Moth-Poulsen
Institute of Materials Science of Barcelona, ICMAB-CSIC, Bellaterra, Barcelona, 08193, Spain.

Dr. S. Henninger, G. Munz and M, Knaebbeler-Buss
Heating and Cooling Technologies/Hydrogen Technologies and Electrical Energy Storage
Fraunhofer Institute for Solar Energy systems (ISE)
Heidenhofstr. 2 79110 Freiburg, Germany
Markus.knaebbeler-buss@ise.fraunhofer.de

Contents

Synthesis…………………………………………………………………………………………3

Characterization

Powder X-ray diffraction (PXRD)…………………………………………………………..4

Metal concentration measurement (Atomic Absorption Spectroscopy)………………..6

Scanning electron microscopy (SEM)……………………………………………………..8

Diffuse reflectance infrared Fourier transform spectroscopy (DRIFTS)………...…….11

Surface area measurement (Nitrogen physisorption)…………………………………..15

Dynamic vapor sorption (DVS)……………………………………………………………18

Differential scanning calorimetry (DSC)…………………………………………………19

Nuclear magnetic resonance (NMR)……………………………………………………..23

Results & Testing

Batch reactions……………………………………………………………………………..25

Reaction kinetics……………………………………………………………………………27

References………………………………………………………………………………….29

Synthesis

The Pt-Ox, Cu-Ox and Ni-Ox supported (nominal 5.0 ±0.5.-wt% metal) activated carbon catalyst was synthesized by the conventional impregnation method (i.e., incipient wetness method). A Norit SX Plus activated carbon was initially washed in deionised water and dried overnight at 120 °C in a Nabertherm muffle oven. Here, 95.0 mg of activated carbon was weighed in and a 5 weight% of metal was added for each catalyst. 13.3 mg of dihydrogen platinum chloride hexahydrate precursor, 19.0 mg of copper nitrate trihydrate and 28.0 mg of nickel acetate tetrahydrate were used. The pore volume of the Norit SX Plus activated carbon is 605 µL/g, thus, to fill the exact pore volume, each metal precursor was dissolved in 57.5 µL of ethanol (apart from nickel acetate tetrahydrate which was dissolved in deionised water), sonication was used to aid dissolution. The precursor solution was added dropwise using a syringe pump at a rate of 10 µL/min to the carbon support. The sample was mixed thoroughly prior to placing it in the muffle oven. Firstly, a ramp in temperature was applied from 25 °C – 120 °C at 60 °C/hour, the temperature was held at 120 °C for 2 hours, next a ramp in temperature from 120 °C – 300 °C at 60 °C/hour was done and finally the samples were calcined at 300 °C for 12 hours. The sample was cooled to room temperature and stored in an air-tight vial prior to use.

The Pt-Red, Cu-Red and Ni-Red supported activated carbon catalyst was synthesized by the conventional impregnation method (i.e., incipient wetness method). All details of the impregnation method remained the same as described for the oxidised versions. The calcination procedure was performed in a tubular reactor where the samples were placed in a ceramic tray. Firstly, a ramp up in temperature was applied from 25 °C – 120 °C at 60 °C/hour, the temperature was held at 120 °C for 2 hours.

The Pt-Red and Ni-Red samples were calcined at 300 °C under nitrogen flow (100 mL/min) for 12 hours. Next, a hydrogen flow (100 mL/min) was applied for two hours maintaining a temperature of 300 °C to do the reduction. The samples were cooled to room temperature in a nitrogen flow of 100 mL/min and stored in an air-tight vial prior to use.

The Cu-Red sample was calcined at 300 °C under argon flow (100 mL/min) for 12 hours. Next, a hydrogen flow (100 mL/min) was applied for two hours maintaining a temperature of 300 °C. The samples were cooled to room temperature in a nitrogen flow of 100 mL/min and stored in an air-tight vial prior to use.

Characterization

Powder X-Ray Diffraction (PXRD)

Structural phase identification of Pt-Ox, Cu-Ox, Ni-Ox, Pt-Red, Cu-Red, and Ni-Red catalysts were recorded using a Rigaku XRD Diffractometer Mini Flex 600 equipped with a Cu kα radiation with a generator voltage and a current of 45 kV and 40 mA, respectively. The measurements were carried out in a 2θ range of 10-90°, using a step size of 0.05° and a counting time of 0.5 seconds per step.

Scherrer-Debye equation:

$$L = K \lambda / \beta \cos\Theta \qquad \textit{Equation 1}$$

Where K is the Scherrer constant – 0.94 (assuming spherical particles), λ – wavelength of the X-ray beam used in nm – 0.154, β – full width at half maximum height of the peak - in radians, Θ - Bragg's angle of diffraction, L – crystallite size.

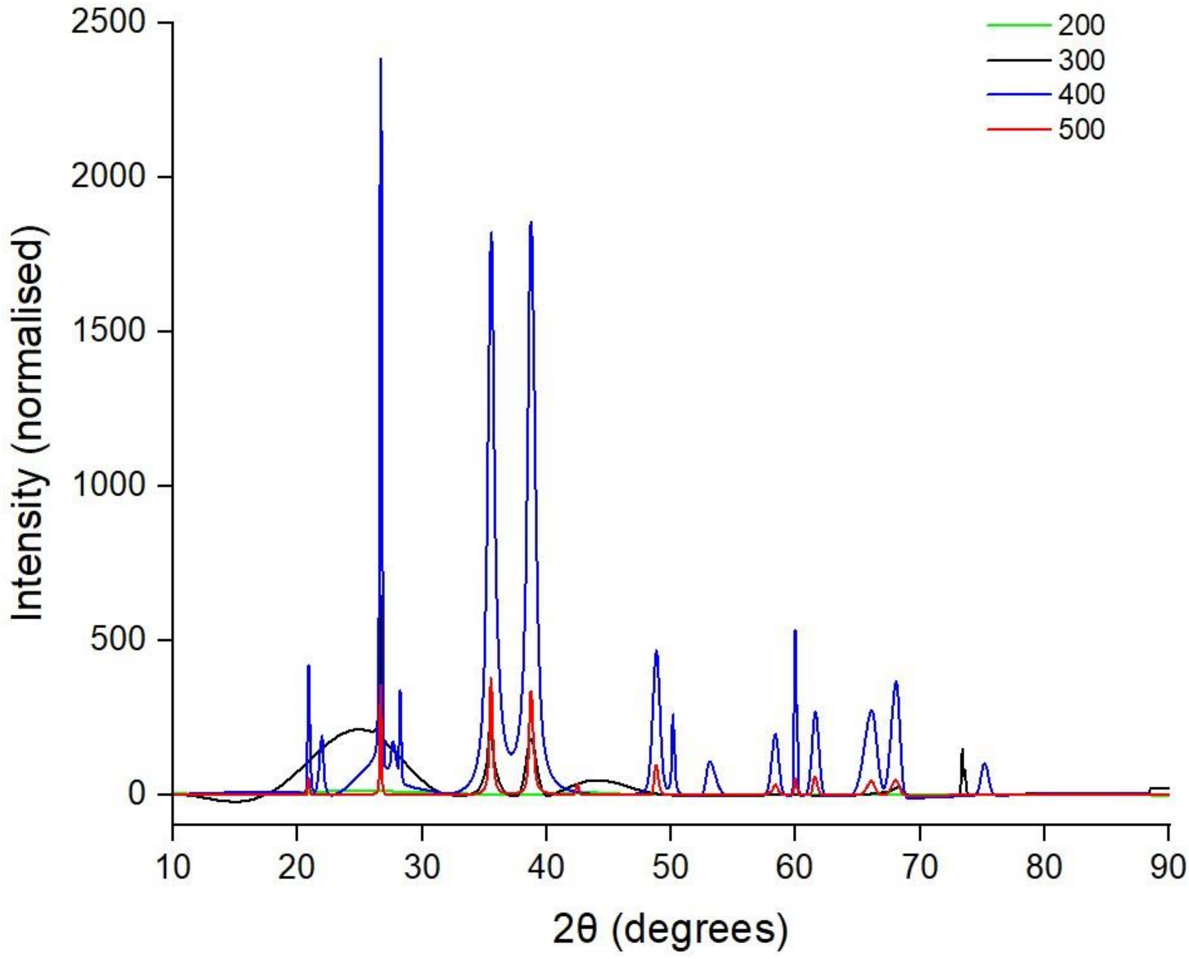

Figure S1 PXRD graphs of Cu-Ox catalyst calcinated at various temperatures (°C).

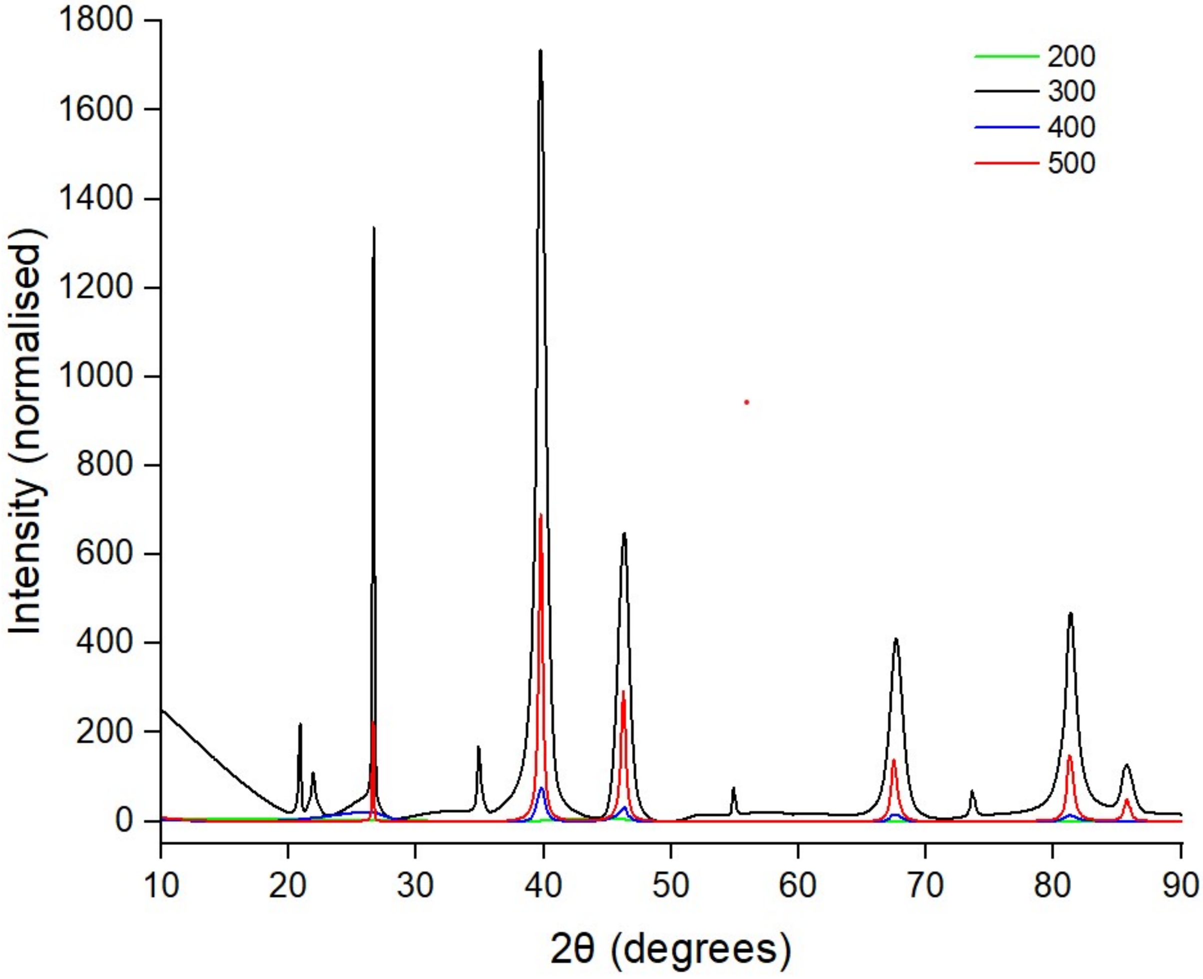

Figure S2 PXRD graphs of Pt-Ox catalyst calcinated at various temperatures (°C).

Metal concentration measurement – Atomic Absorption Spectroscopy (AAS)

A PinAAcle 500 Flame Atomic Absorption Spectrometer instrument was used to measure the total metal loading of the prepared catalysts. A 2.5 mg mass of catalyst sample was digested in aqua regia under reflux for 48 hours and the extract was diluted by a factor of 10 prior to analysis. NIST traceable standards of Pt, Cu, Ni were used to obtain the calibration graphs.

When calculating the weight % just follow this procedure:

Digest 2.5 milligrams of catalyst in 10 mL of aqua regia (1-part nitric acid: 3 parts hydrochloric acid). Once digested, take a 1 mL aliquot from the digested solution, and dilute by 10 mL to be in the range of the AAS instrument. The recorded values after interpolation using the calibration curves gives the concentration of metal in solution in ppm or mg of metal/Litre.

*mg of Metal/mg = ((X mg/L * (10 mL / 1 mL) * 0.01 L) / 2.5 mg) x 100*   *Equation 2*

Table S1 The absorbance values experimentally measured using AAS for each catalyst, wherein concentrations were calculated through interpolation of the calibration curves and the weight % was calculated from equation 2.

Catalyst	Absorbance (a.u)	Concentration (mg/L)	Weight %
**Pt-Red**	0.00260	1.460	5.838
**Pt-Ox**	0.00466	2.016	8.063
**Cu-Red**	0.0654	1.646	6.583
**Cu-Ox**	0.0596	1.471	5.884
**Ni-Red**	0.296	2.022	8.086
**Ni-Ox**	0.268	1.686	6.745

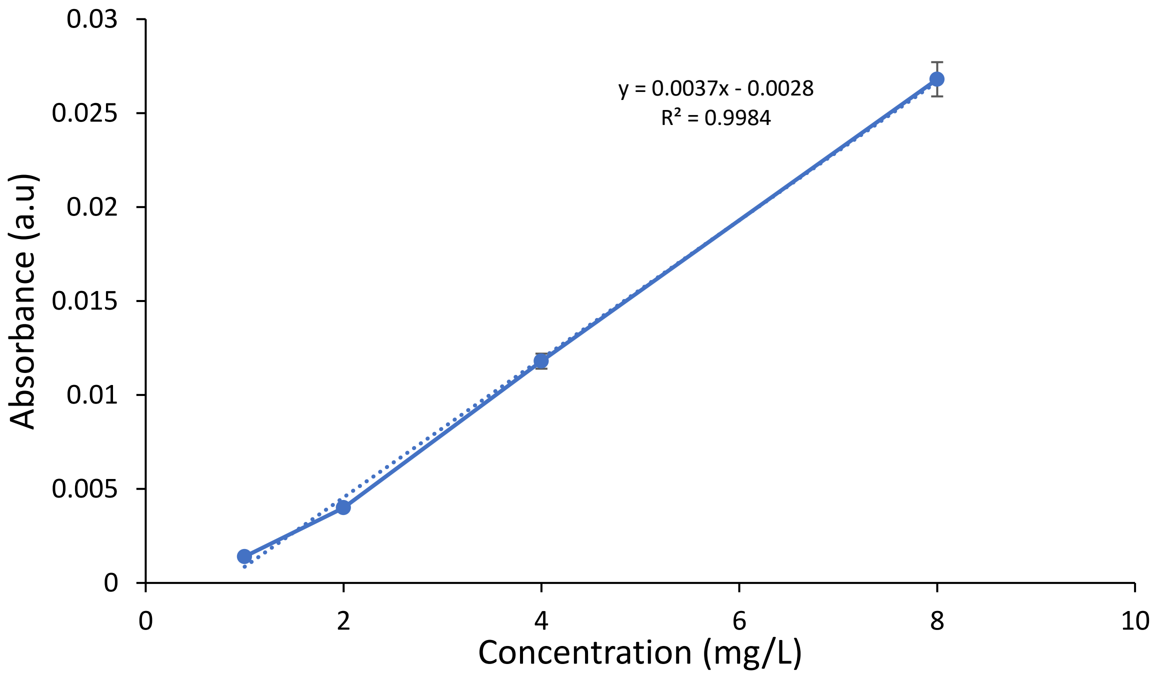

Figure S3 Calibration curve for platinum.

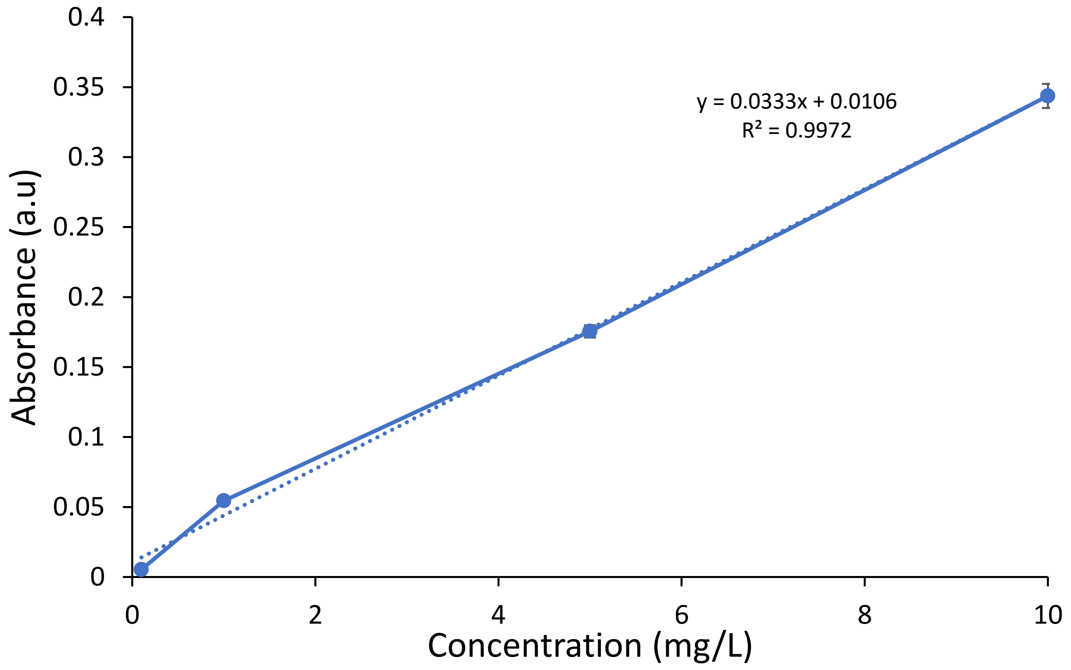

Figure S5 Calibration curve for copper.

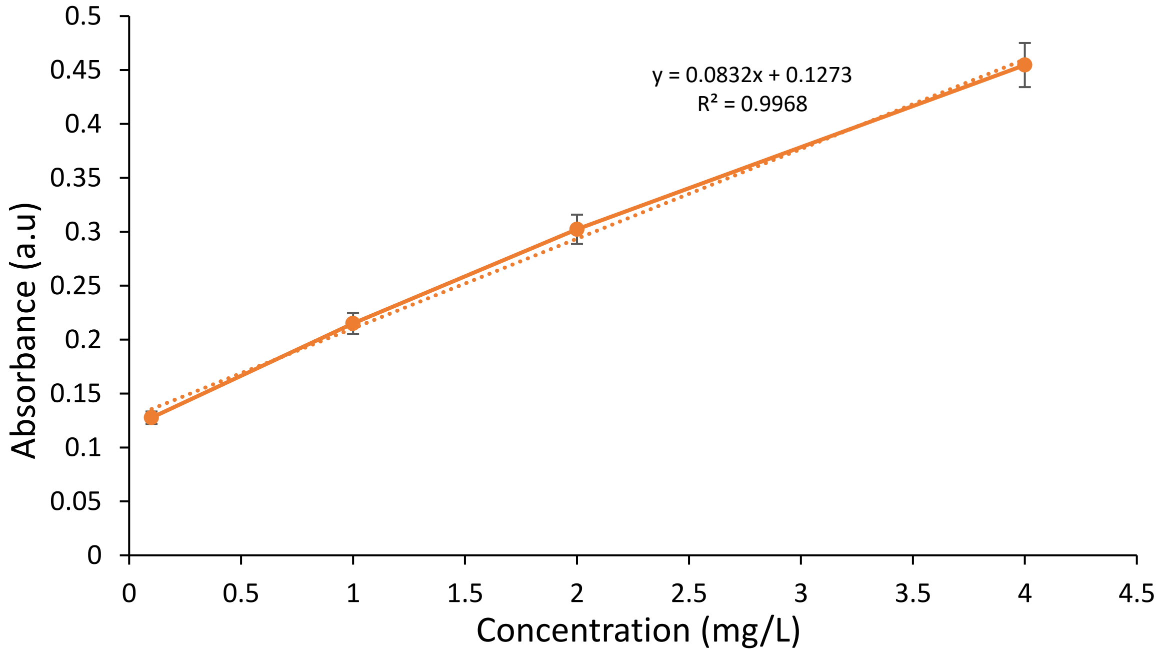

Figure S4 Calibration curve for nickel.

Scanning electron microscopy (SEM)

To understand the morphology of the catalysts, high-resolution scanning electron microscopy images were taken using a Zeiss Gemini SEM 360 instrument. Sample preparation was done by applying a high purity double sided adhesive conductive carbon tape to the SEM stub. Next, the catalyst powder was gently covered over the SEM stub, where remaining particles was removed using a gentle flow of nitrogen gas.

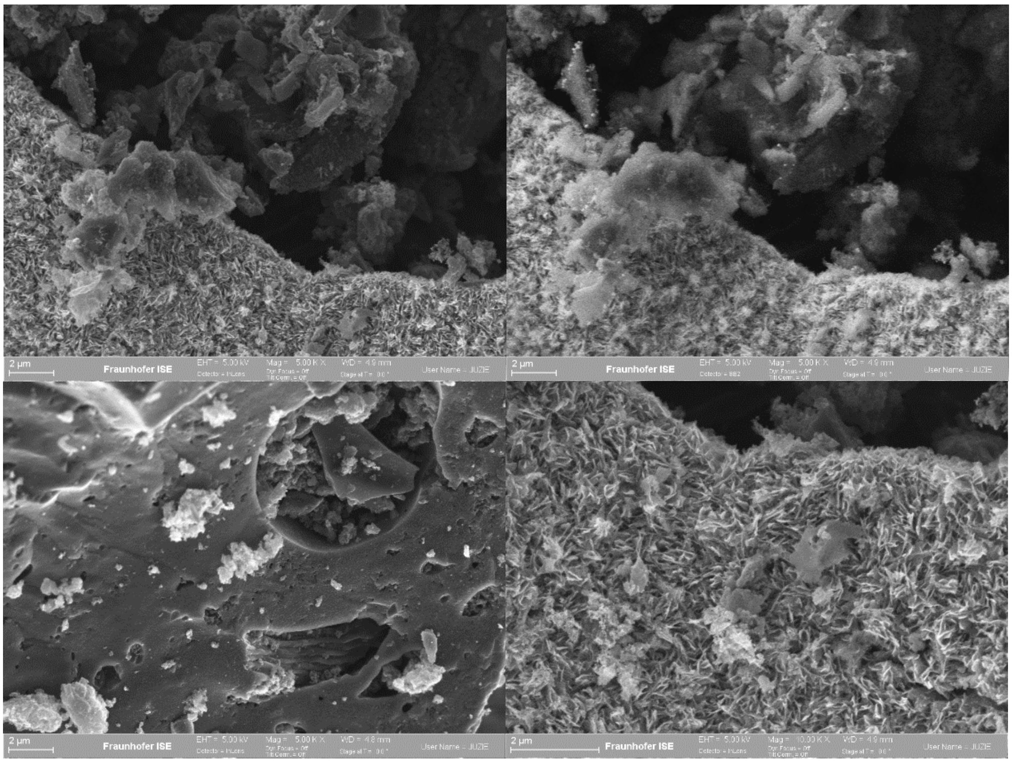

Figure S6 Ni-Red catalyst: top left - inLens, top right - SE2, bottom left - inLens, bottom right – inLens.

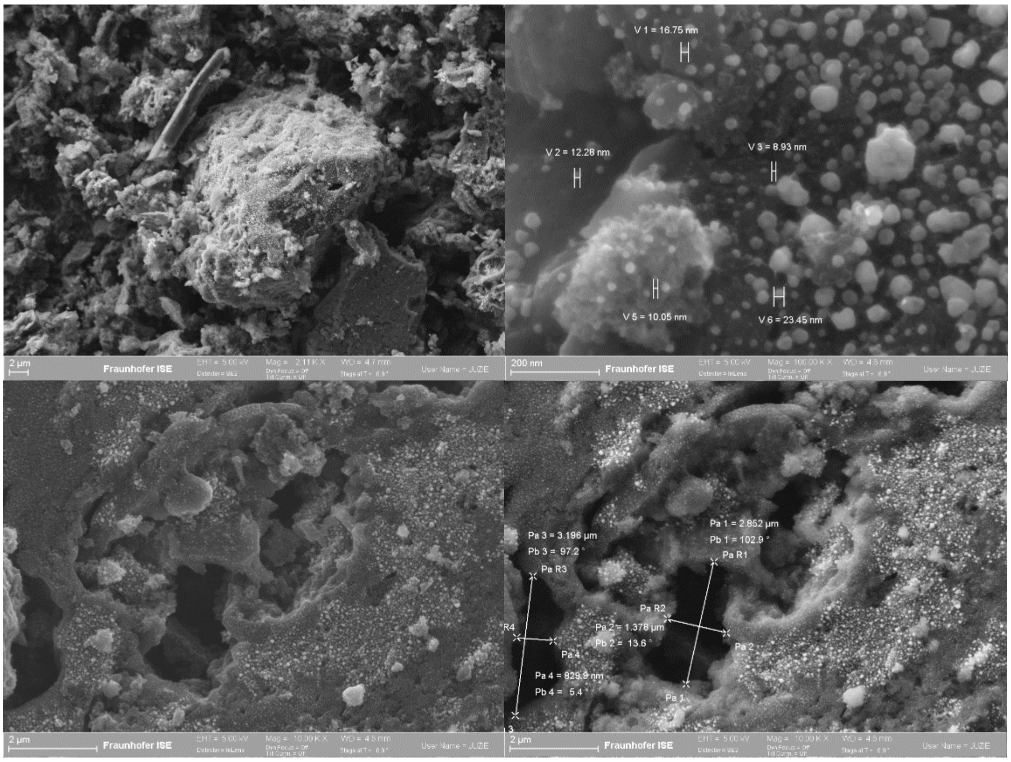

Figure S7 Cu-Red catalyst: top left – SE2, top right – inLens, bottom left – inLens, bottom right – SE2.

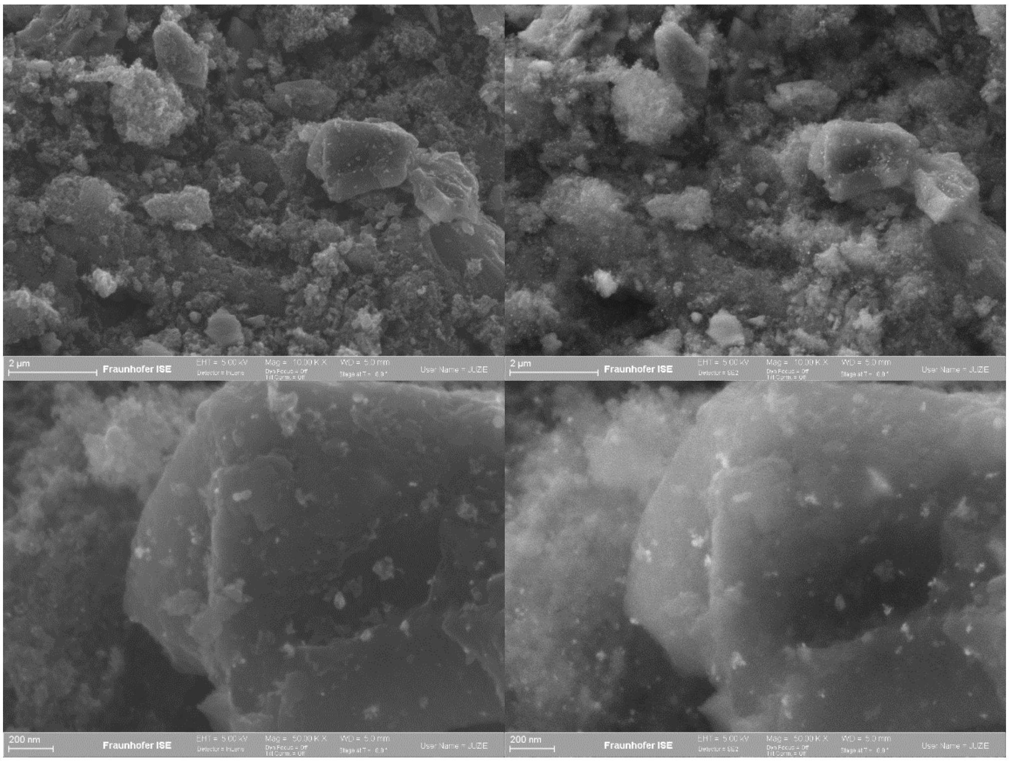

Figure S8 Pt-Red catalyst: top left – inLens, top right – SE2, bottom left – inLens, bottom right – SE2.

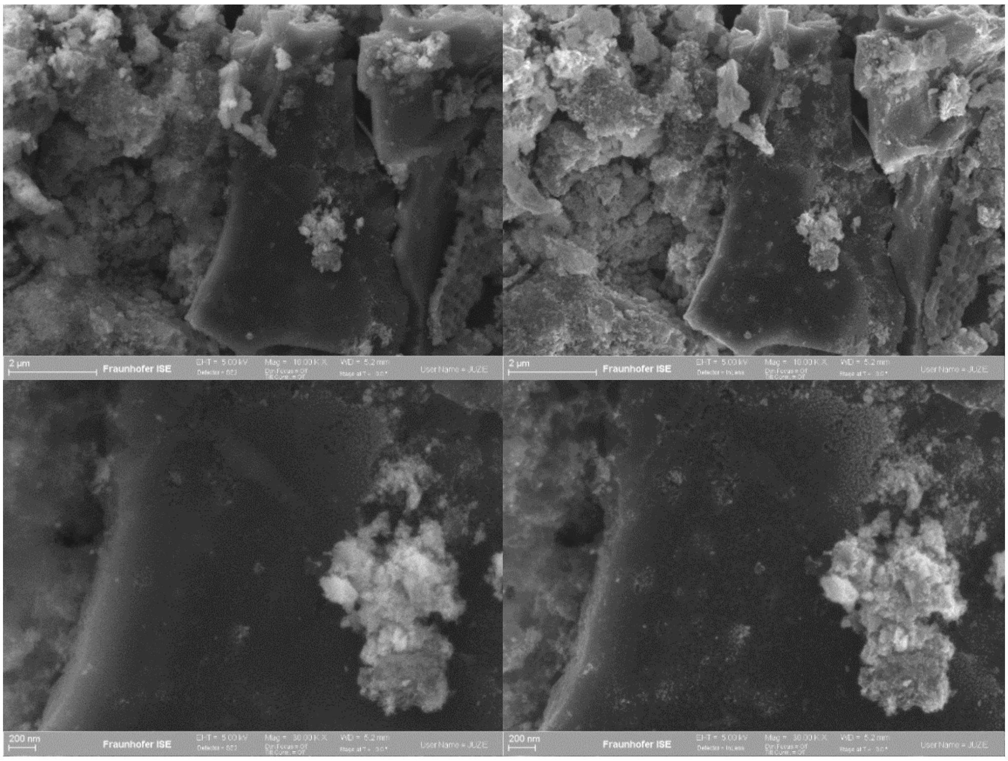

Figure S9 Ni-Ox catalyst: top left – SE2, top right – inLens, bottom left – SE2, bottom right – inLens.

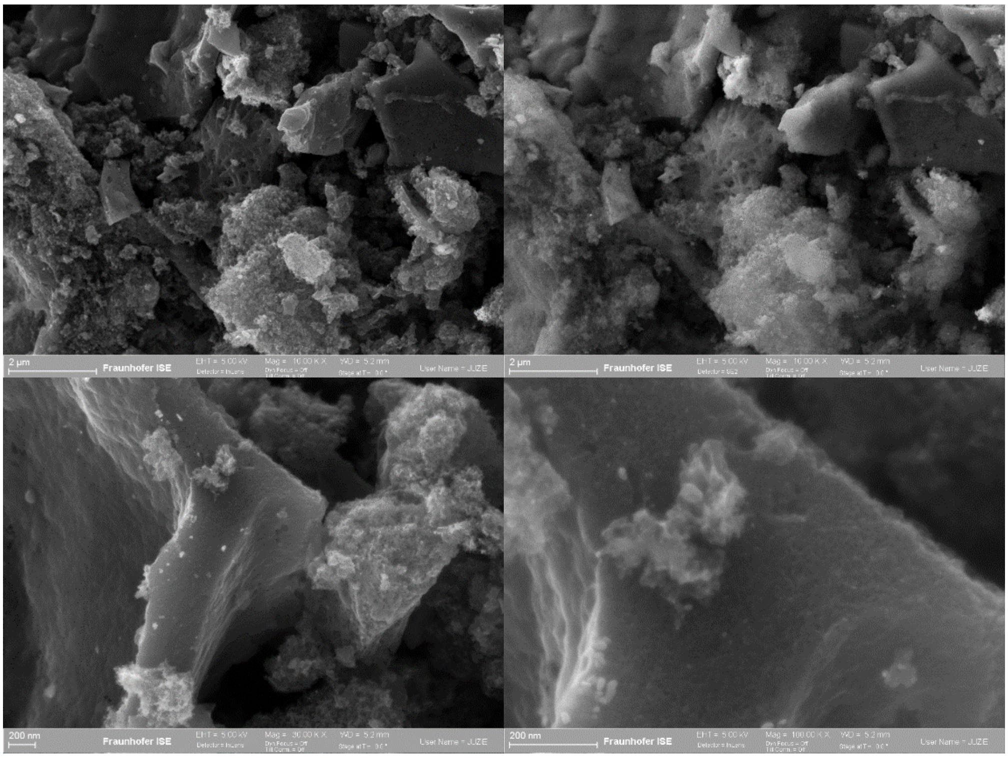

Figure S10 Cu-Ox catalyst: top left – inLens, top right – SE2, bottom left – inLens, bottom right – inLens.

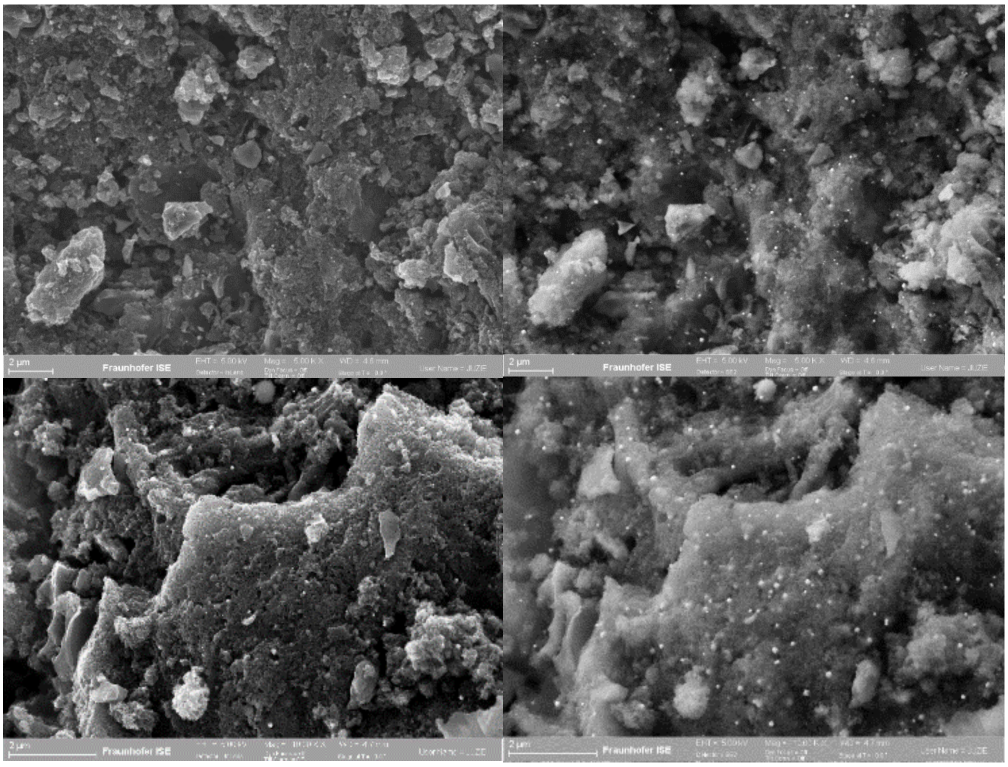

Figure S11 Pt-Ox catalyst: top left - inLens, top right - SE2, bottom left - inLens, bottom right – SE2.

Diffuse reflectance infrared Fourier transform spectroscopy (DRIFTS)

DRIFTS spectra were obtained in the range of 400 cm^{-1} – 4000 cm^{-1} after six scans with a resolution of 4 cm^{-1} by using a Perkin Elmer Lambda Spectrum Two FTIR instrument. A ceramic holder with sample pressed flat was placed in a DRIFTS cell with a quartz window. Prior to analysis, the DRIFTS cell was flushed using flowing nitrogen gas for 5 minutes. All the samples were measured using a weight ratio of 1:100 KBr: catalyst, where KBr was used as an irradiating matrix.

Table S2 Assignment of functional groups common in a variety of activated carbon materials.

**Functionality**	**Assignment regions (cm^{-1})**		
	**1000-1500**	**1500-2050**	**2050-3700**
C-O in ethers (stretching)	1000-1300		
Alcohols	1049-1276		3200-3640
Phenolic groups:			
C-OH (stretching)	1000-1220		
OH	1160-1200		2500-3620
Carbonates; carboxyl-carbonates	1100-1500	1590-1600	
C=C aromatic (stretching)		1585-1600	
Quinones		1550-1680	
Carboxylic acids	1120-1200	1665-1760	2500-3300
Lactones	1160-1370	1675-1790	
Carboxylic anhydrides	980-1300	1740-1880	
C-H (stretching)			2600-3000

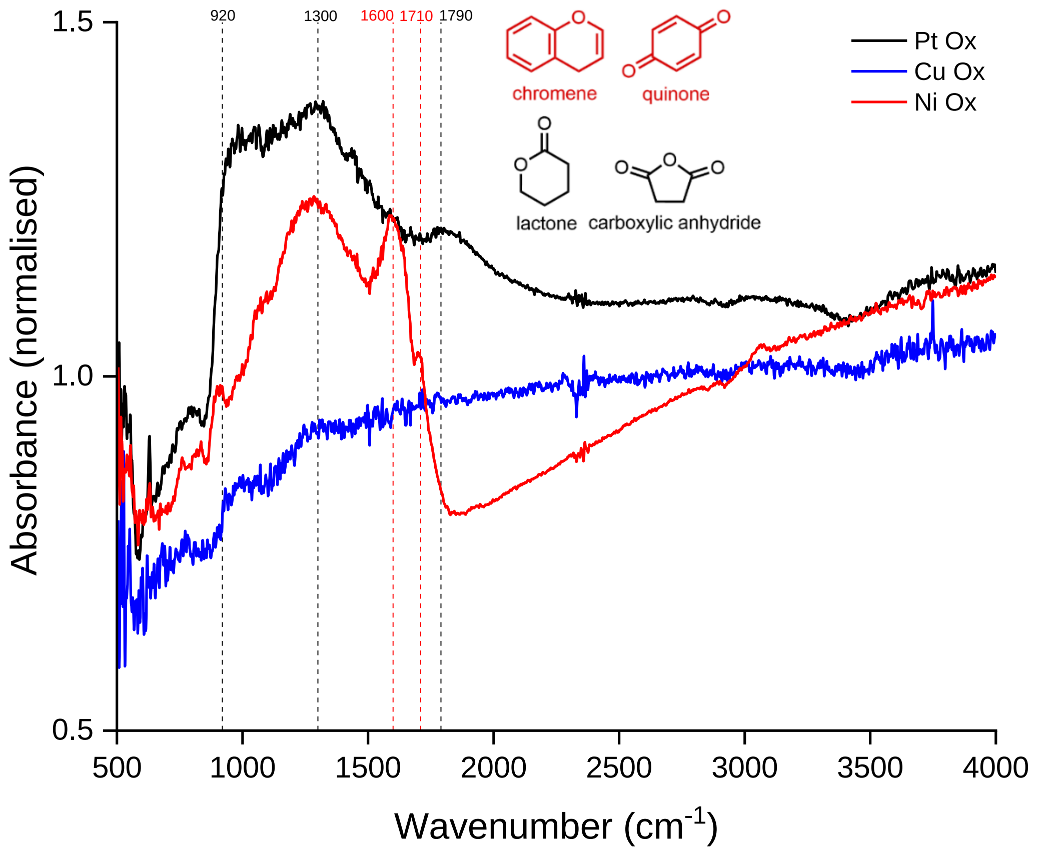

Figure S12 DRIFTS of Pt-Ox, Cu-Ox and Ni-Ox catalysts measured from 500 cm^{-1}- 4000 cm^{-1} with six accumulations and a resolution of 4 cm^{-1} using a ratio of 1:100 KBr: catalyst.

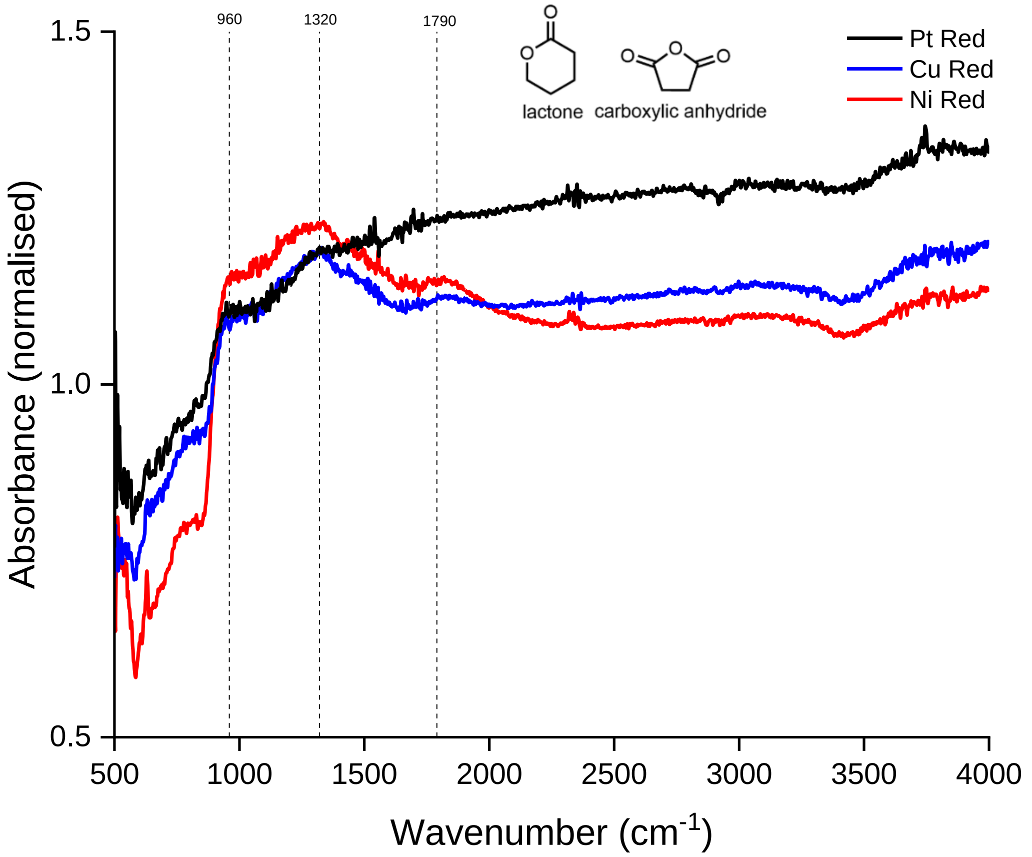

Figure S13 DRIFTS of Pt-Red, Cu-Red, and Ni-Red catalysts measured from 500 cm^{-1}- 4000 cm^{-1} with six accumulations and a resolution of 4 cm^{-1} using a ratio of 1:100 KBr: catalyst.

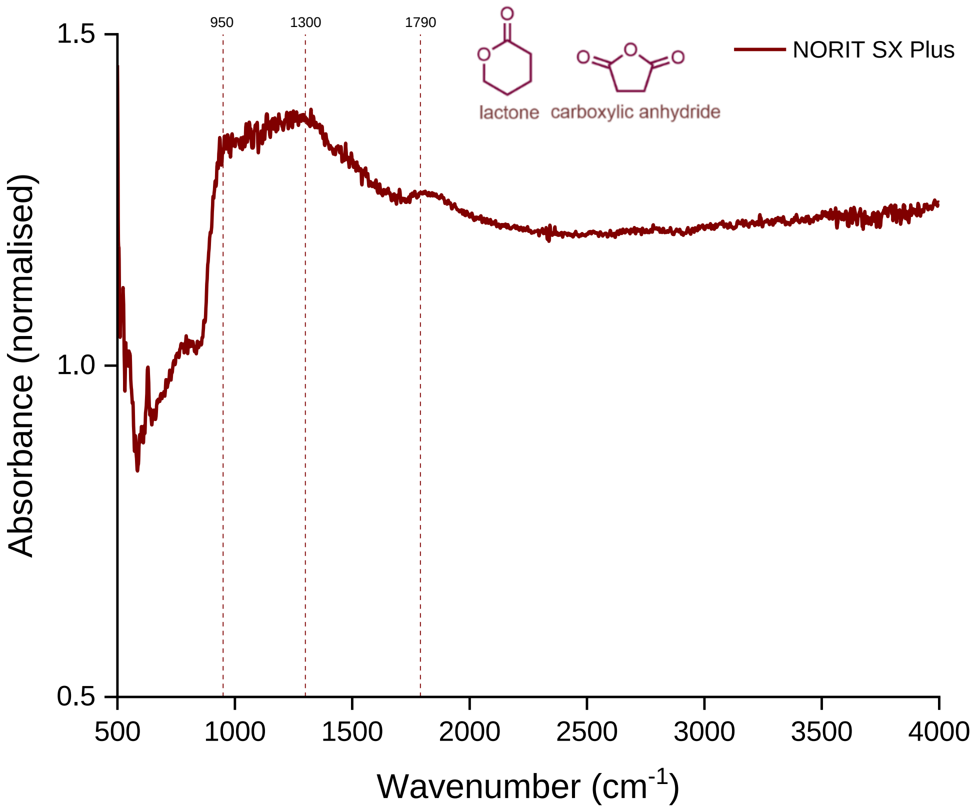

Figure S14 Norit SX Plus activated carbon measured from 500 cm^{-1}- 4000 cm^{-1} with six accumulations and a resolution of 4 cm^{-1} using a ratio of 1:100 KBr: catalyst.

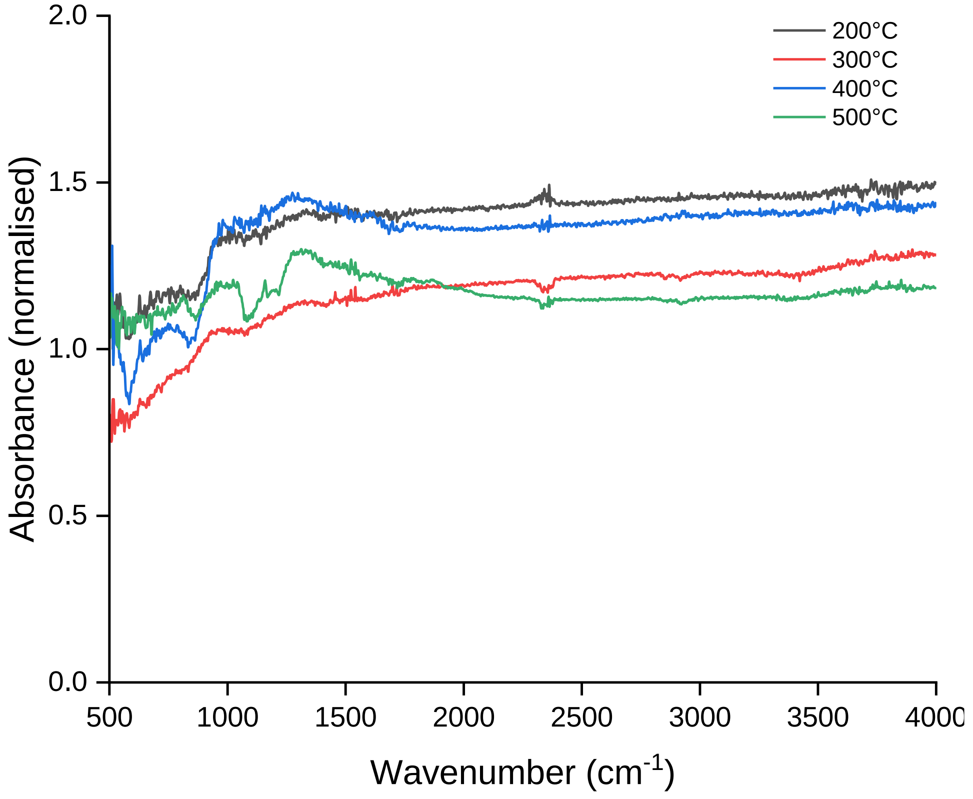

Figure S15 DRIFTS for Pt-Ox catalysts calcinated at 200 °C, 300 °C, 400 °C and 500 °C.

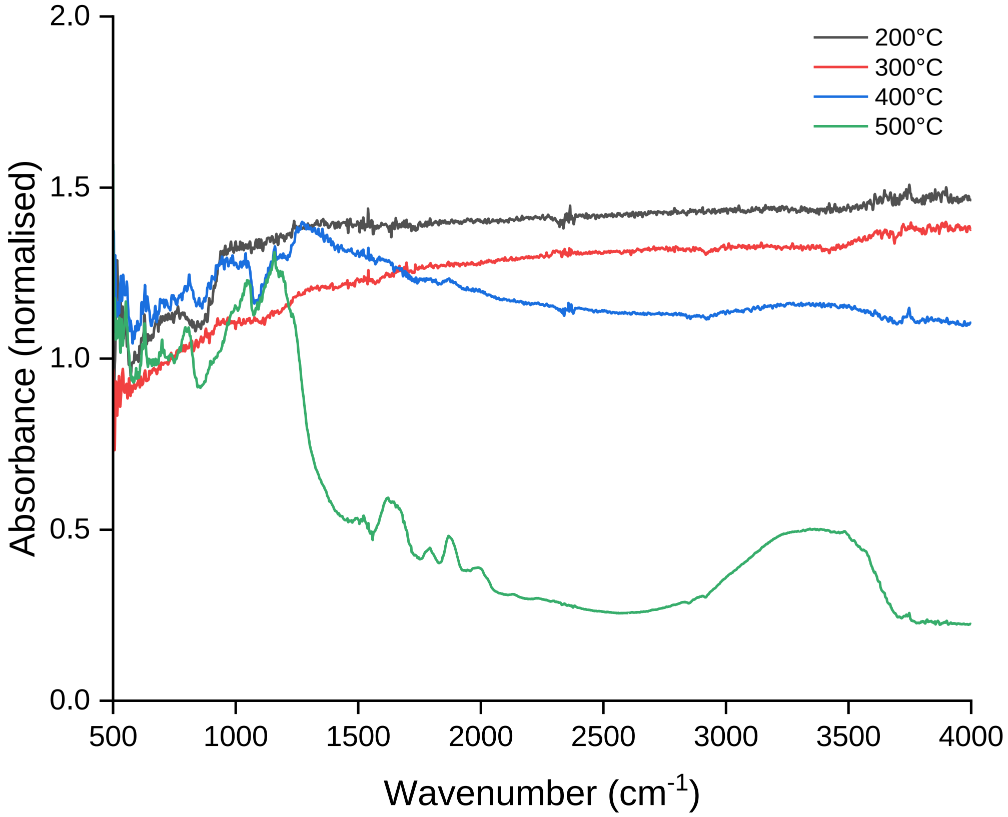

Figure S16 DRIFTS for Cu-Ox catalysts calcinated at 200 °C, 300 °C, 400 °C and 500 °C.

Surface area measurement (nitrogen physisorpton)

Prior to adsorption analysis, removal of impurities from the catalyst surface was performed using an Anton Paar QuantaTec MasterPrep Degasser instrument. The catalyst samples were degassed using nitrogen and heated to 200 °C for 20 hours using a ramp rate of 5 K/min. Nitrogen adsorption–desorption measurements were performed on an Anton Paar Quadrawin instrument at 77 K. The Brunauer-Emmett-Teller (BET) method was applied in a relative pressure range of 0.04 – 1 for the evaluation of the specific surface area and the total pore volume was evaluated from the amount of adsorbed nitrogen at a relative pressure (P/P_0) of about 0.98. The pore diameter distributions were calculated based on desorption isotherms by the Barrett–Joyner– Halenda (BJH) method.

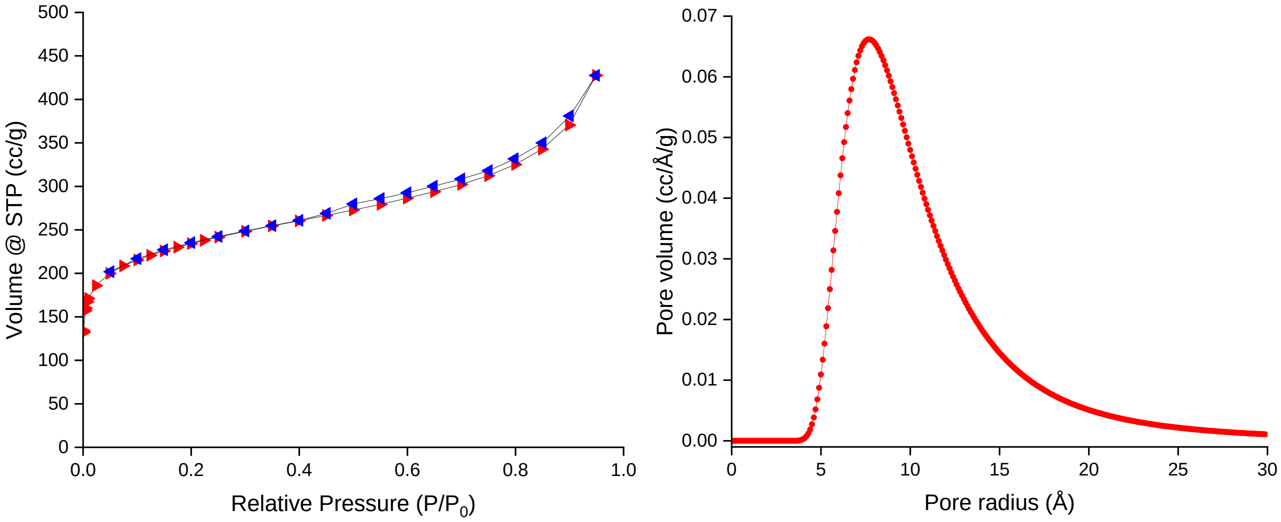

Figure 17 Ni-Red adsorption and desorption isotherm (left) and pore size and volume distribution (right)

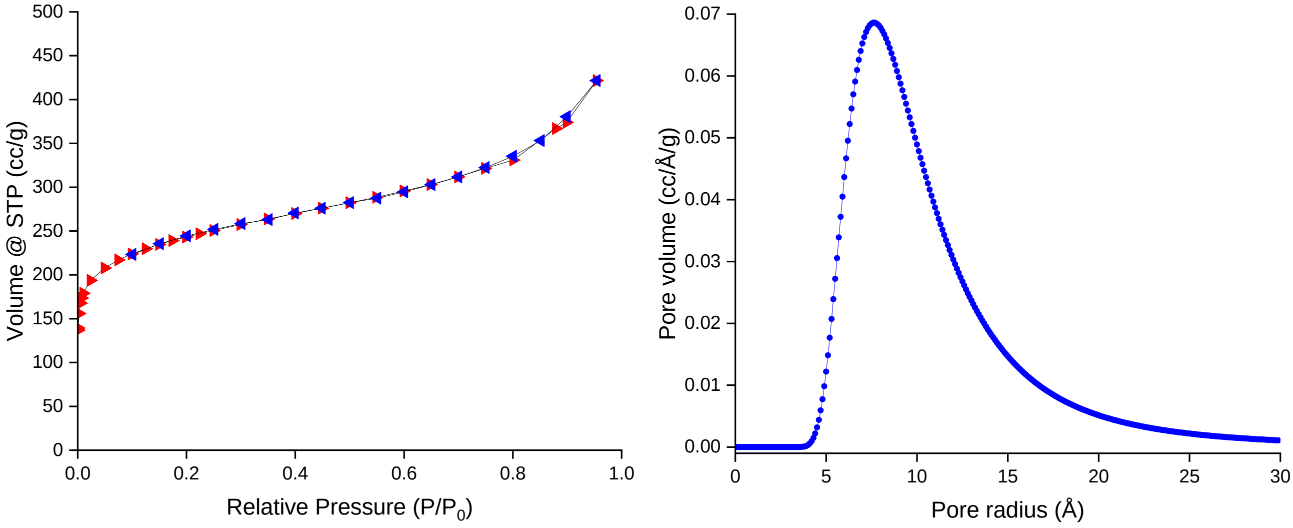

Figure S18 Cu-Red adsorption and desorption isotherm (left) and pore size and volume distribution (right)

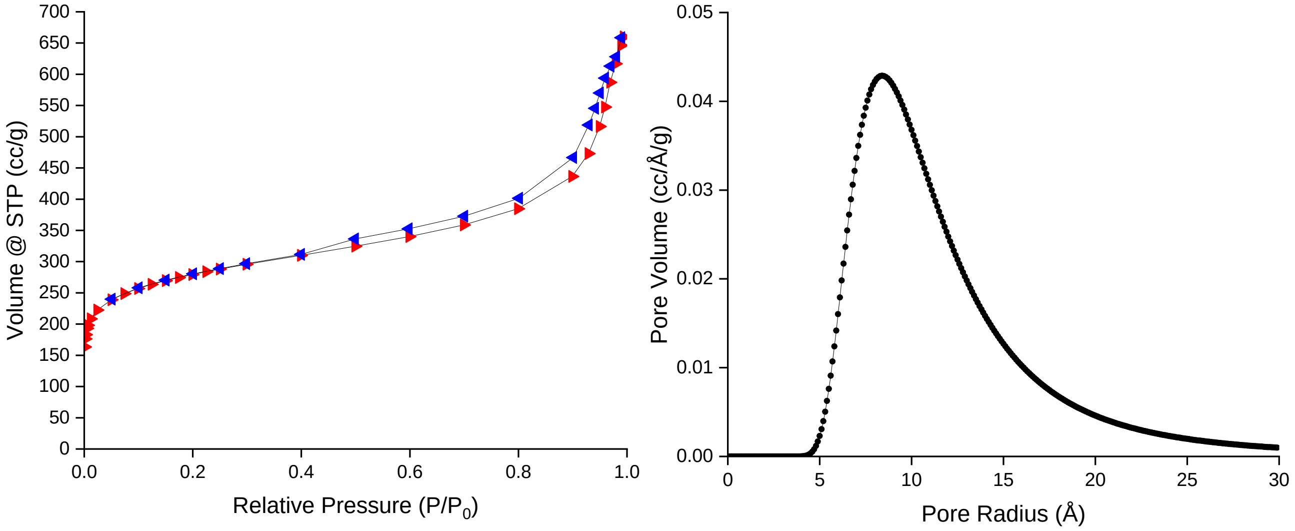

Figure S19 Pt-Red adsorption and desorption isotherm (left) and pore size and volume distribution (right)

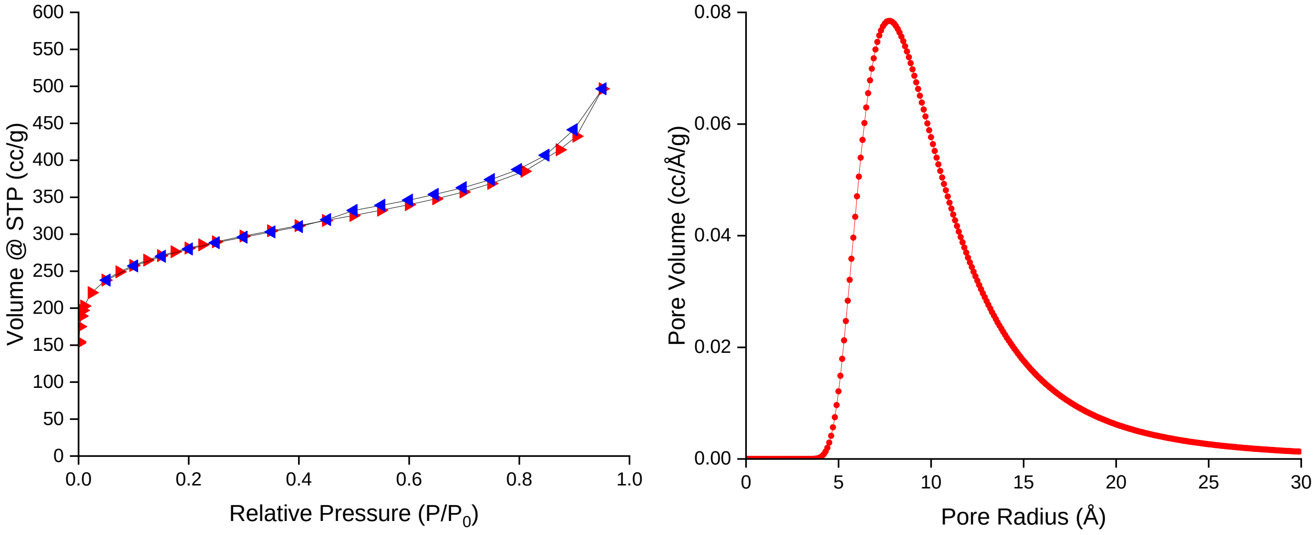

Figure S20 Ni-Ox adsorption and desorption isotherm (left) and pore size and volume distribution (right)

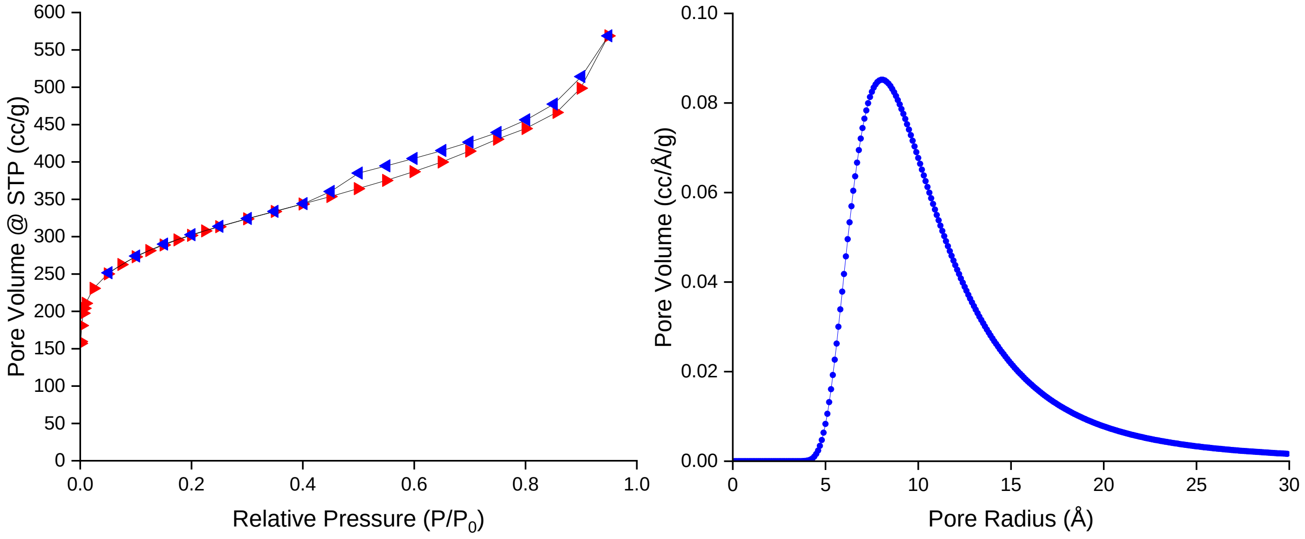

Figure S21 Cu-Ox adsorption and desorption isotherm (left) and pore size and volume distribution (right)

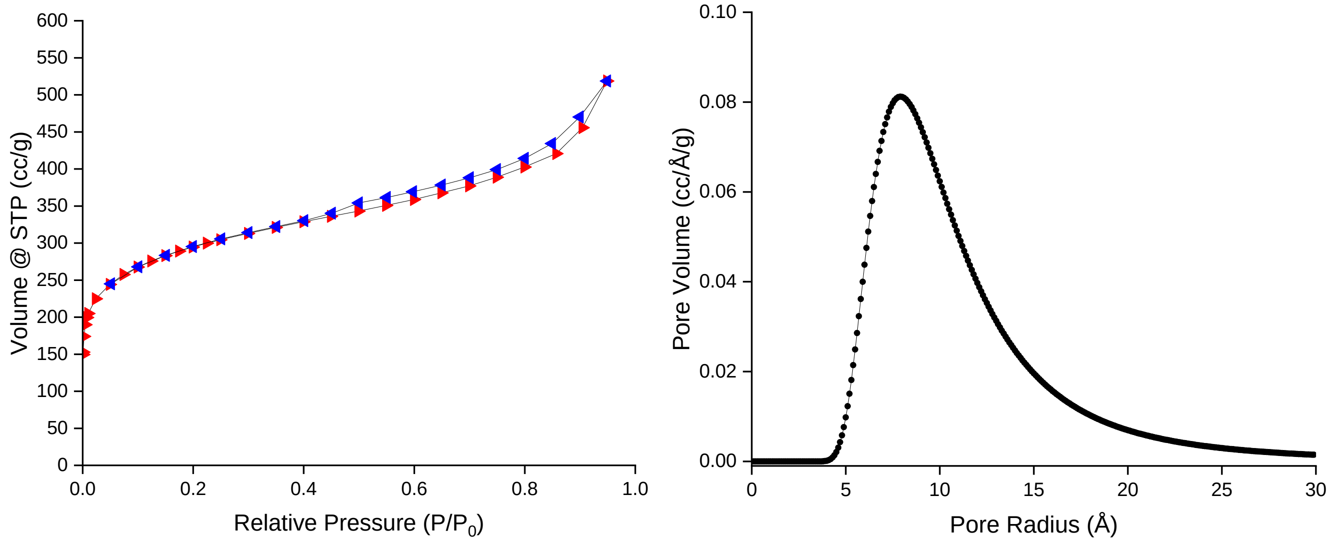

Figure S22 Pt-Ox adsorption and desorption isotherm (left) and pore size and volume distribution (right)

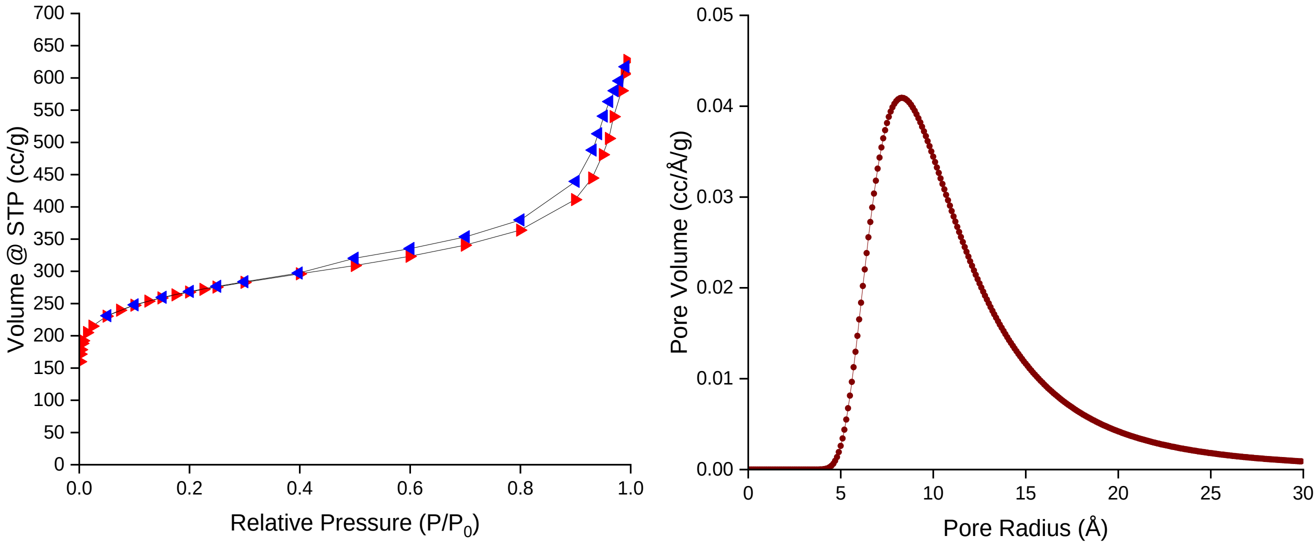

Figure S23 Norit SX Plus adsorption and desorption isotherm (left) and pore size and volume distribution (right)

Dynamic vapour sorption (DVS)

Prior to adsorption analysis, removal of impurities from the catalyst surface was performed using an Anton Paar QuantaTec MasterPrep Degasser instrument. The catalyst samples were degassed using nitrogen and heated to 200 °C for 20 hours using a ramp rate of 5 K/min. The vapour sorption isotherms of the oxidised and reduced catalysts were measured using an Anton Paar QuantaTec Vstar instrument. The measurement was performed over a relative pressure range of 0.001 – 1.

Differential scanning calorimetry (DSC)

Heating and cooling measurements were performed using a Mettler-Toledo (DSC2A-00312) instrument. The sample was loaded into a Tzero^{TM} aluminum hermetic pan, and the measurement was performed in nitrogen atmosphere. The samples were equilibrated at 40 °C for 2 minutes, a ramp-up to 400 °C at a heating rate of 3 K/min was used (on the first run a heating rate of 7 K/min) and held isothermally for 2 minutes. Next, a ramp-down in temperature to 40 °C at a cooling rate of 3 K/min was done and then held isothermally for 2 minutes. Subsequently, a second ramp-down to -90 °C was completed and held isothermally for 2 minutes. A second cycle up to 400 °C was repeated and held isothermally for 2 minutes.

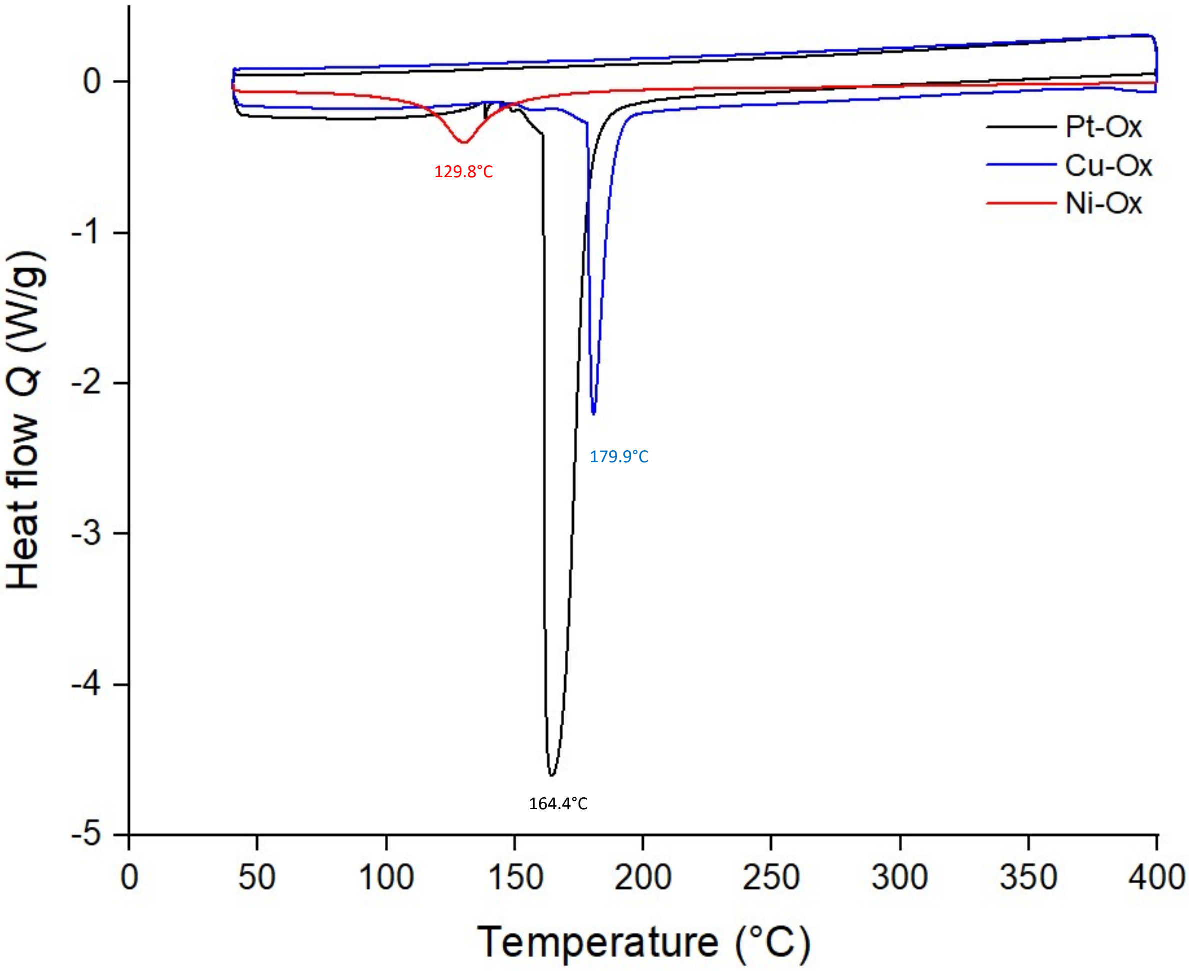

Figure S24 Heating and cooling cycle of Pt-Ox, Cu-Ox and Ni-Ox catalysts at a ramp rate of 3 K/min.

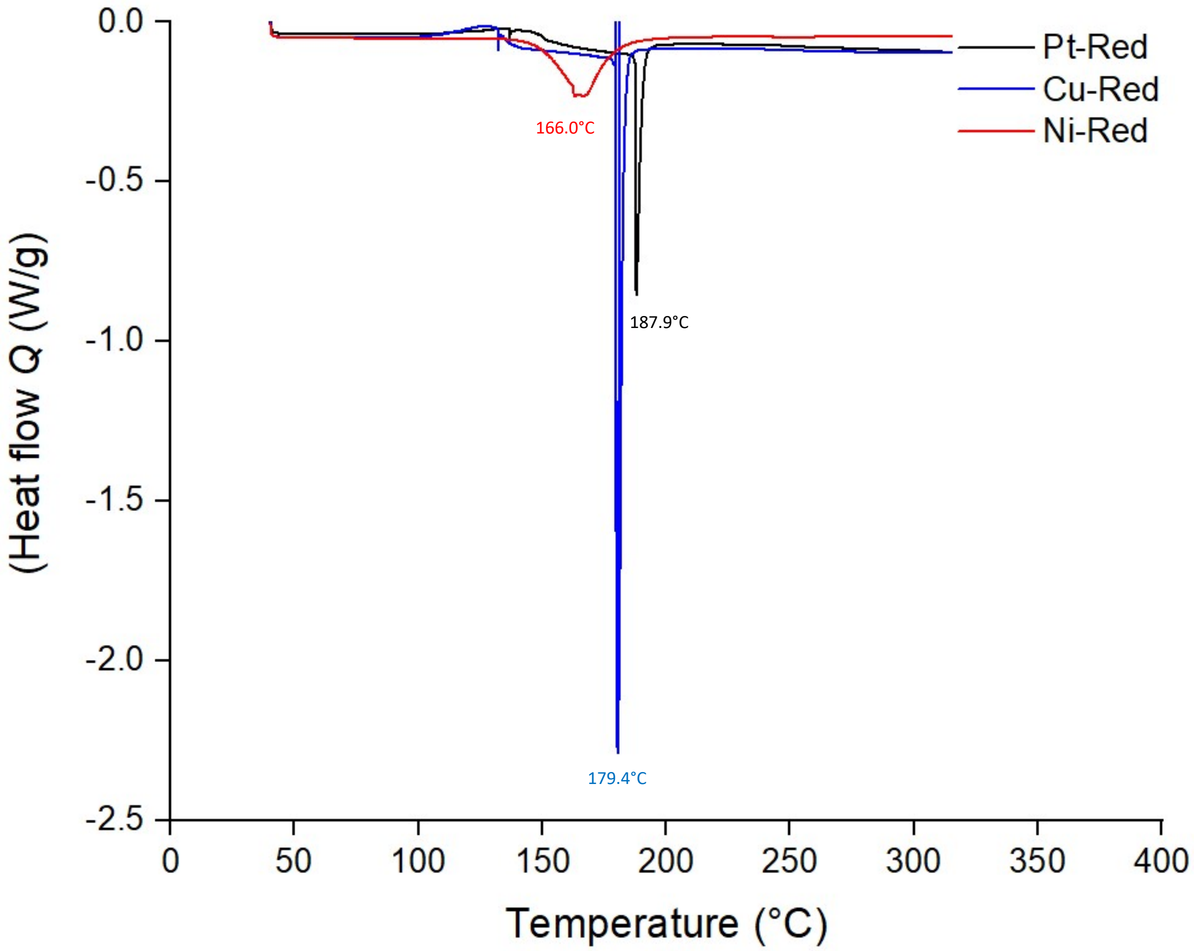

Figure S25 Heating and cooling cycle of Pt-Ox, Cu-Ox and Ni-Ox catalysts at a ramp rate of 3 K/min.

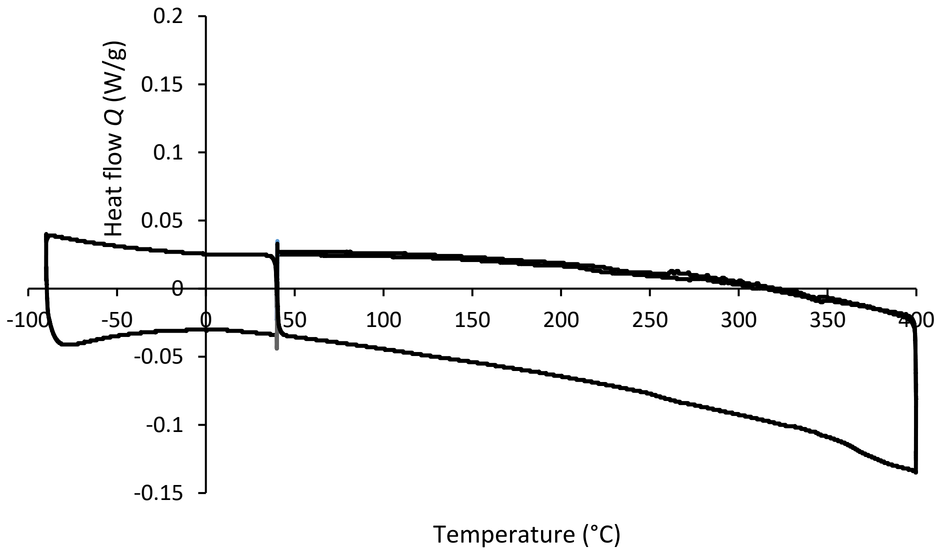

Figure S26 Second heating and cooling cycle of the Pt-Ox catalyst at a ramp rate of 3 K/min.

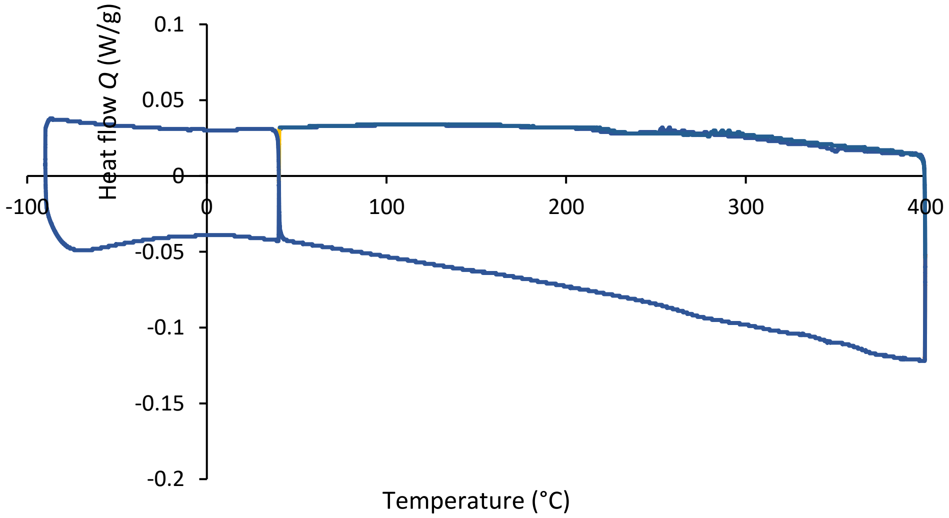

Figure S27 Second heating and cooling cycle of the Cu-Ox catalyst at a ramp rate of 3 K/min.

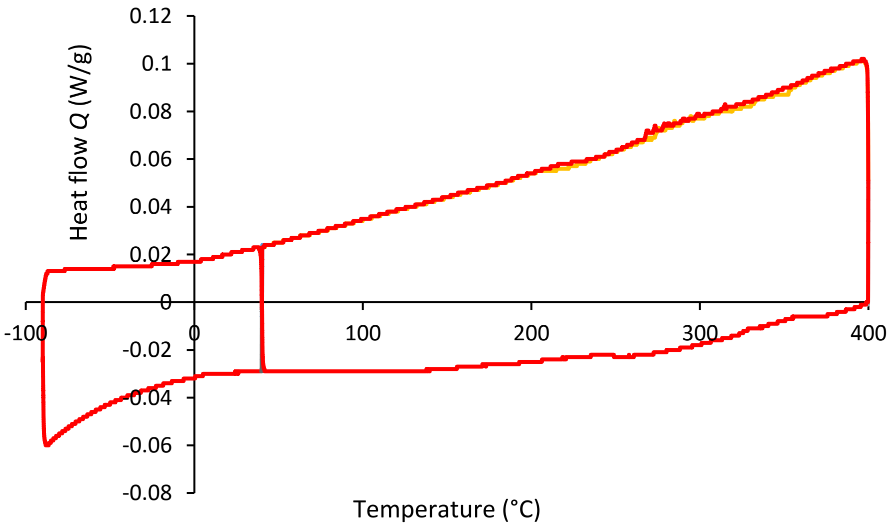

Figure S28 Second heating and cooling cycle of the Ni-Ox catalyst at a ramp rate of 3 K/min.

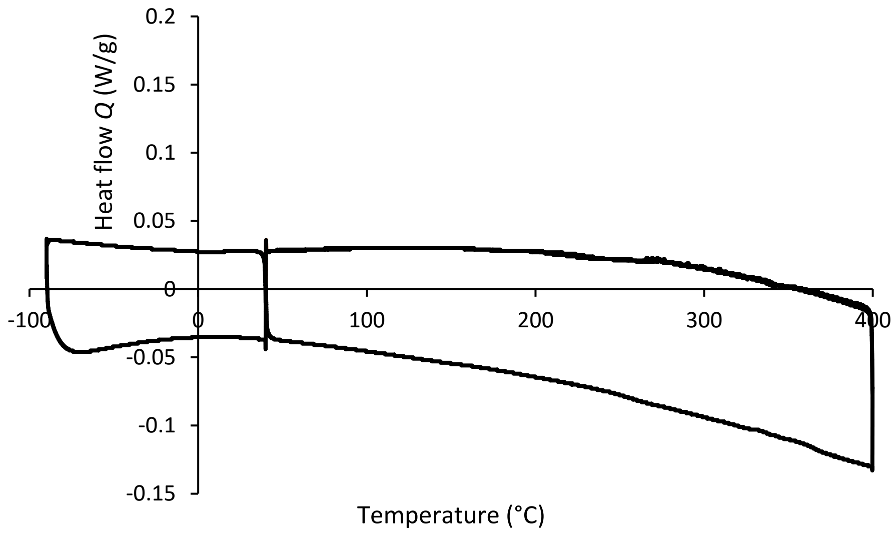

Figure S29 Second heating and cooling cycle of the Pt-Red catalyst at a ramp rate of 3 K/min.

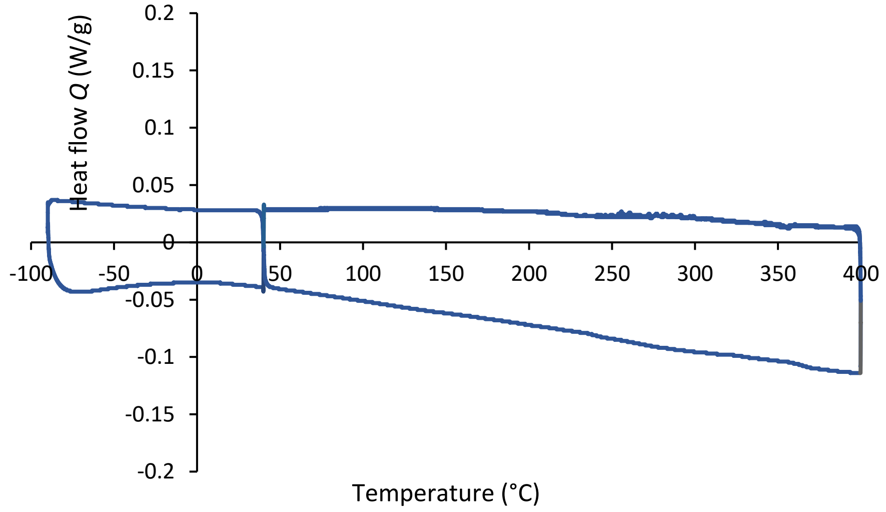

Figure S30 Second heating and cooling cycle of the Cu-Red catalyst at a ramp rate of 3 K/min.

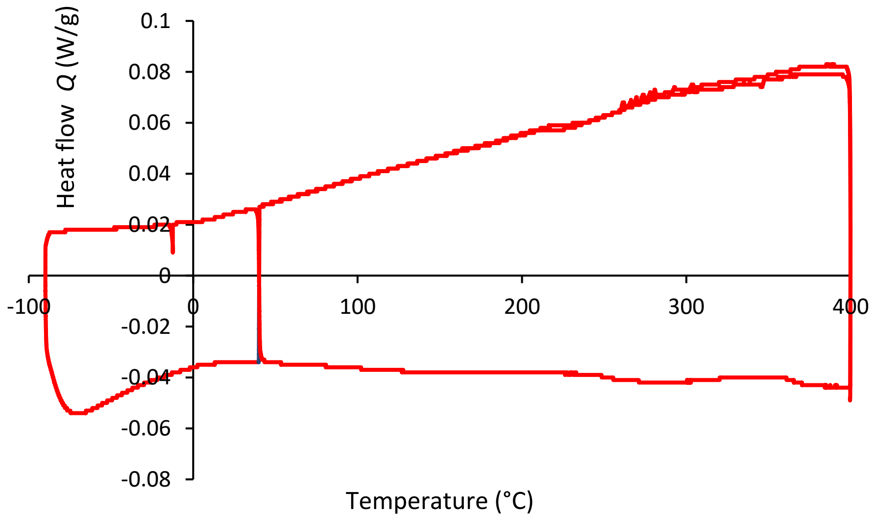

Figure S31 Second heating and cooling cycle of the Ni-Red catalyst at a ramp rate of 3 K/min.

Nuclear magnetic resonance (NMR)

The NMR spectra were recorded on a Bruker 400 MHz Ultrashield^{TM} spectrometer.

^{1}H NMR (300 MHz, Tol) δ 7.62 (d, 2H), 6.62 (d, 2H), 6.48 (ddd, *J* = 5.1, 2.9, 0.8 Hz, 1H), 6.41 (ddd, *J* = 5.2, 3.1, 0.9 Hz, 1H), 3.57 (ddtd, *J* = 3.2, 2.4, 1.6, 0.7 Hz, 1H), 3.45 (ddtd, *J* = 3.2, 2.7, 1.7, 0.8 Hz, 1H), 3.27 (s, 3H), 1.81 (dt, *J* = 6.7, 1.7 Hz, 1H), 1.74 (dt, *J* = 6.7, 1.6 Hz, 1H) ppm.

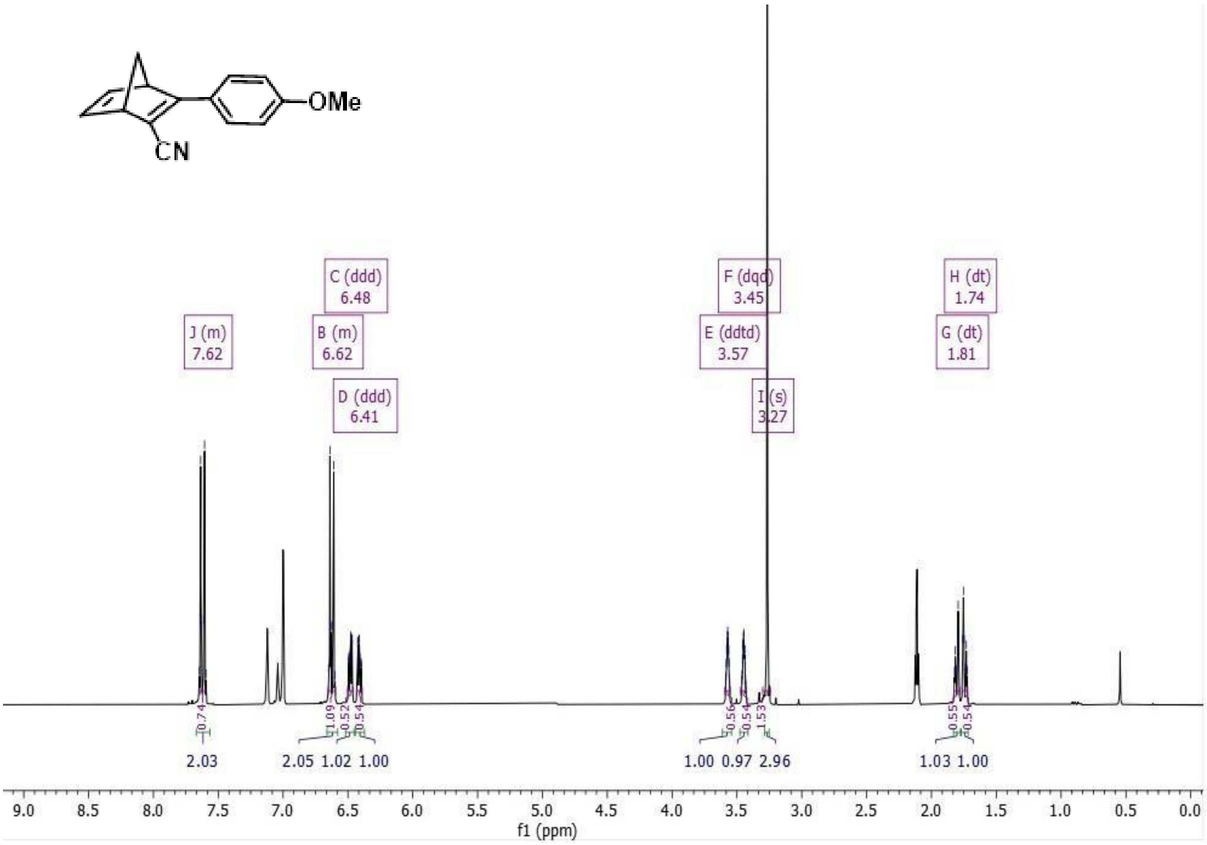

Figure S32 ^{1}H NMR of cyano-3-(4-methoxyphenyl)-norbornadiene.

Synthesis of cyano-3-(4-methoxyphenyl)-norbornadiene/quadricyclane pair was accomplished using a literature procedure.[1]

^{1}H NMR (300 MHz, Tol) δ 7.04 (d, *J* = 8.9 Hz, 2H), 6.68 (d, *J* = 8.8 Hz, 2H), 3.31 (s, 3H), 1.90 – 1.79 (m, 2H), 1.65 (dq, *J* = 4.9, 1.4 Hz, 1H), 1.62 – 1.53 (m, 2H), 1.28 (dq, *J* = 5.0, 1.5 Hz, 1H).

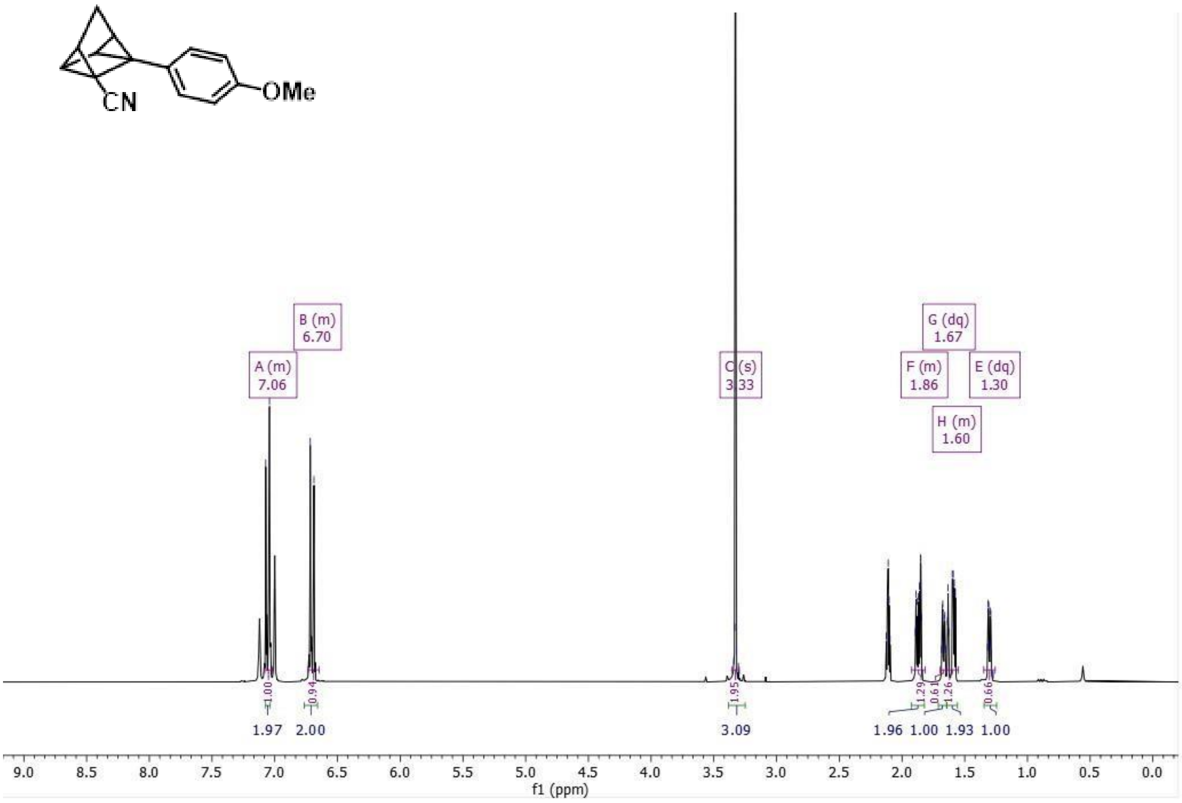

Figure S33 ^{1}H NMR of cyano-3-(4-methoxyphenyl)-quadricyclane.

Results & Testing

Batch reactions

LuzChem photoreactor LZC4 contains 15 UVA lamps of 8 Watts. The UV-Vis measurements were performed on a UV-3600 Shimadzu UV-Vis NIR spectrometer.

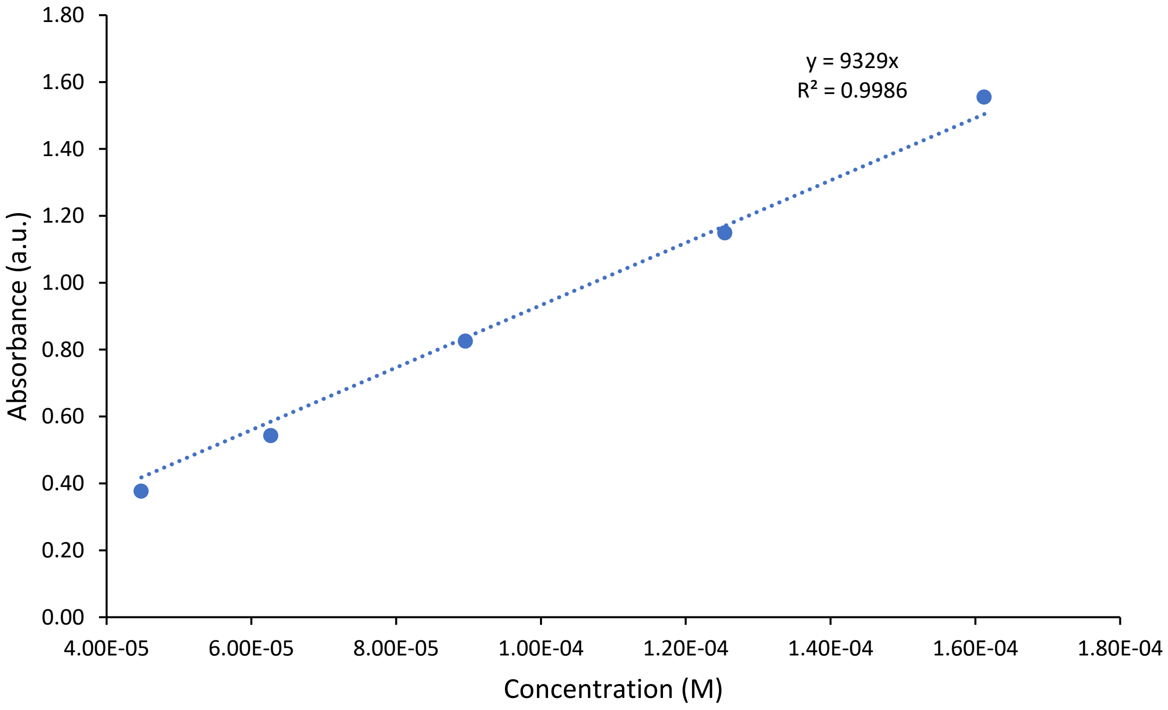

Figure S34 Calibration curve of NBD, absorbance value taken at 340 nm.

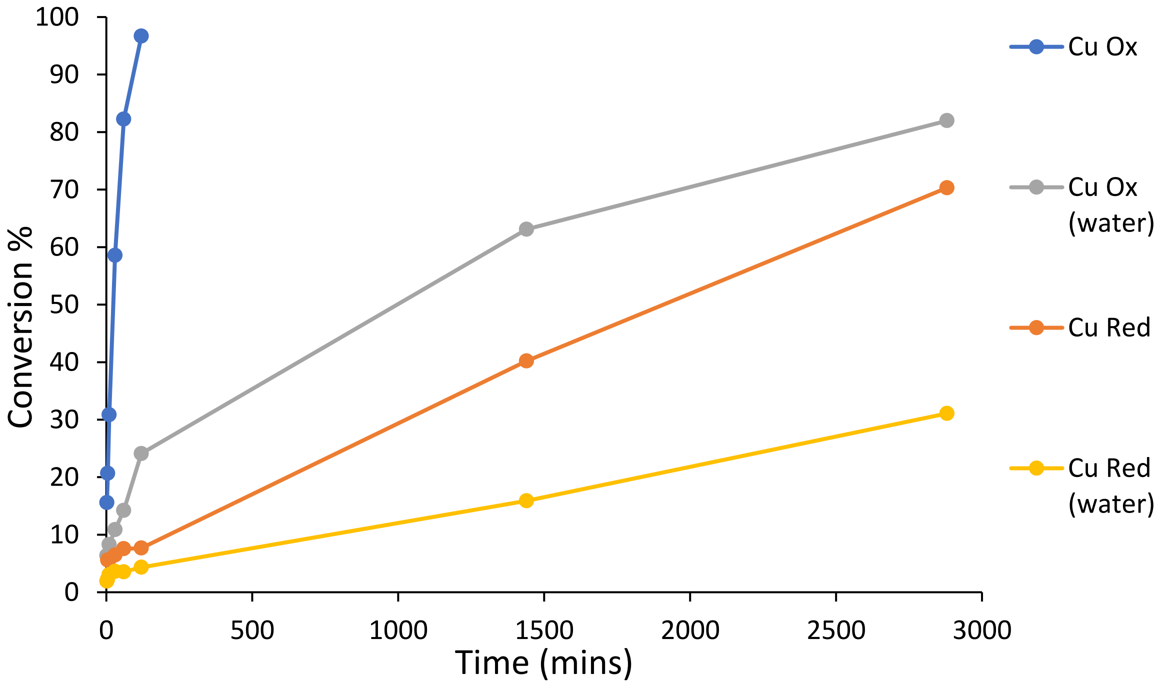

Figure S35 Batch reactivity of Cu-Ox and Cu-Red catalysts soaked in water for 6 hours, then a vacuum pump used to dry the respective catalysts for one day. These are compared to the batch reactivity of the Cu-Ox and Cu-Red catalysts.

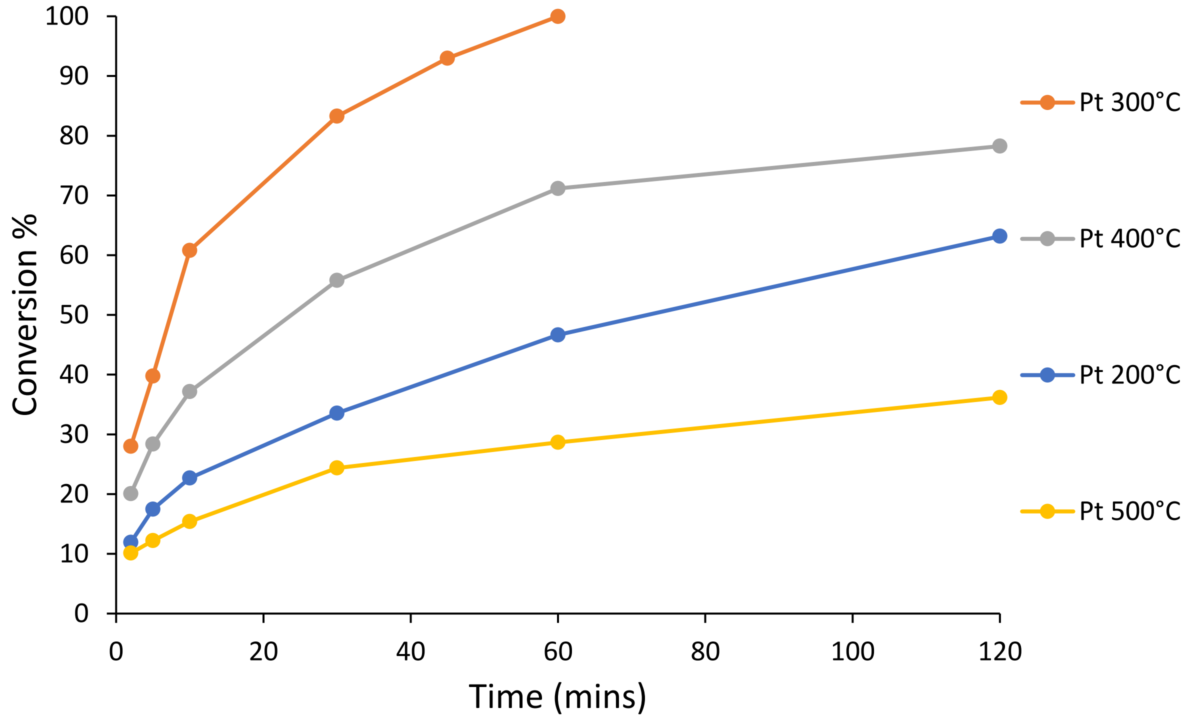

Figure S36 Batch reactivity of Pt-Ox when catalyst is synthesized using different calcination temperatures.

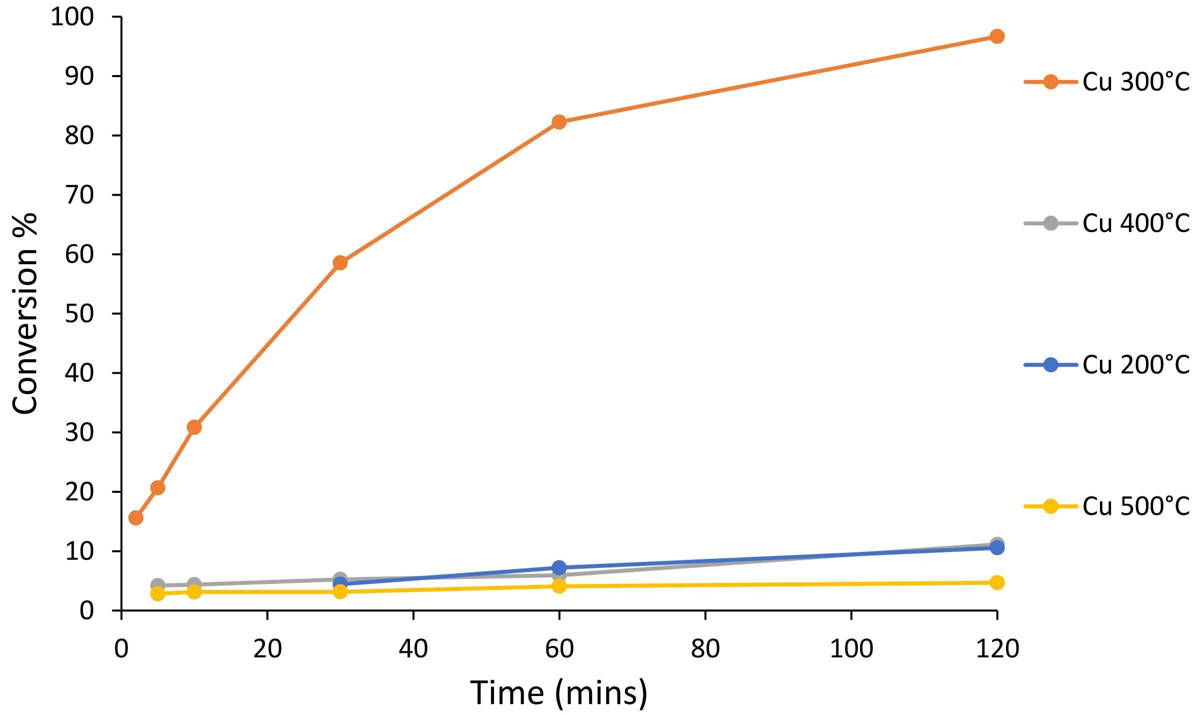

Figure S37 Batch reactivity of Cu-Ox when catalyst is synthesized using different calcination temperatures.

Reaction kinetics

In the set of heterogeneous reactions, the rate constants can be expressed relative to the specific surface area *S* of the catalyst ($m^2\ kg^{-1}$). The rate constants were calculated from the equation below:

$$rNBD, S = -\frac{1}{S}\left(\frac{dn(NBD(0) - NBD(X\ mins)}{dt}\right) = kf(cNBD)\ \text{mol kg m}^{-2}\ \text{s}^{-1} \qquad \textit{Equation 2}$$

Table S3 Reaction rates of the three reduced catalysts relative to their specific surface area was calculated from the first order rate constant.

**Catalyst**	**Specific surface area (m^2/kg)**	**Rate constant (k, s^{-1})**	**Rate constant per surface area (kg m^{-2} s^{-1})**
Pt-Red	0.902	4.19×10^{-4}	3.78×10^{-4}
Cu-Red	0.889	4.07×10^{-4}	3.62×10^{-4}
Ni-Red	0.854	1.30×10^{-4}	1.11×10^{-4}

Table S4 Reaction rates of the three oxidized catalysts relative to their specific surface area was calculated from the first order rate constant.

**Catalyst**	**Specific surface area (m^2/kg)**	**Rate constant (k, s^{-1})**	**Rate constant per surface area (kg m^{-2} s^{-1})**
Pt-Ox	1.07	5.54×10^{-2}	5.93×10^{-2}
Cu-Ox	1.09	2.71×10^{-2}	2.95×10^{-2}
Ni-Ox	1.02	1.23×10^{-3}	1.25×10^{-3}

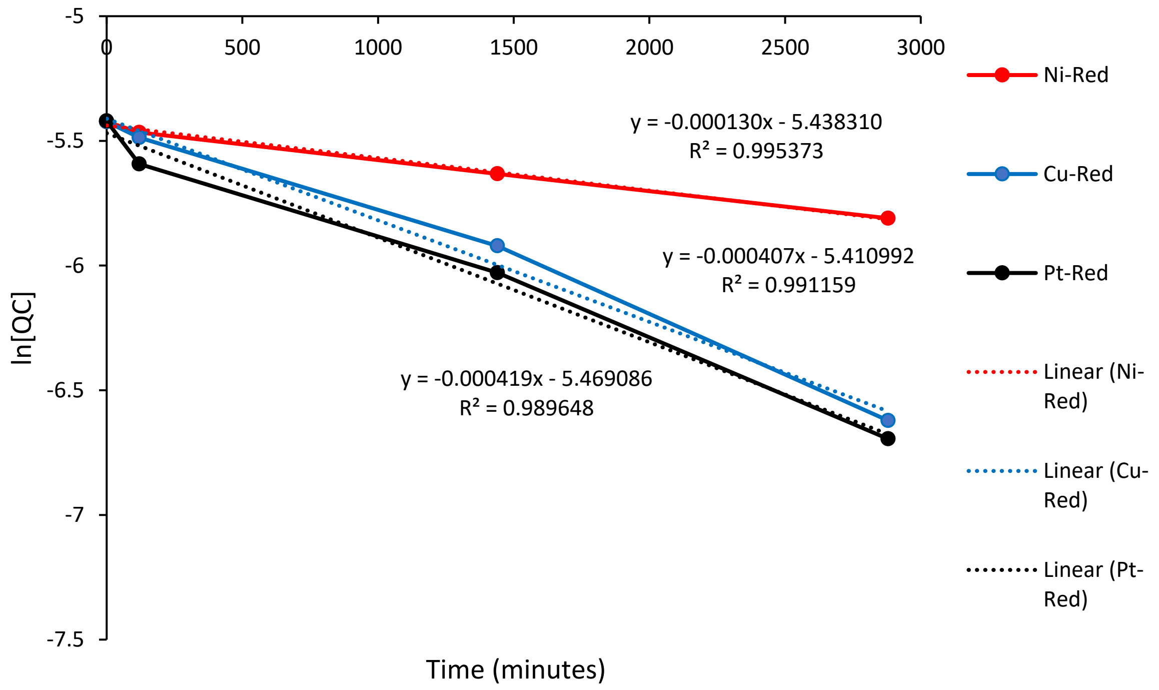

Figure S38 Reaction rates of the catalytic back-reaction from QC to NBD for the reduced catalysts applying the first order kinetics in the logarithmic form.

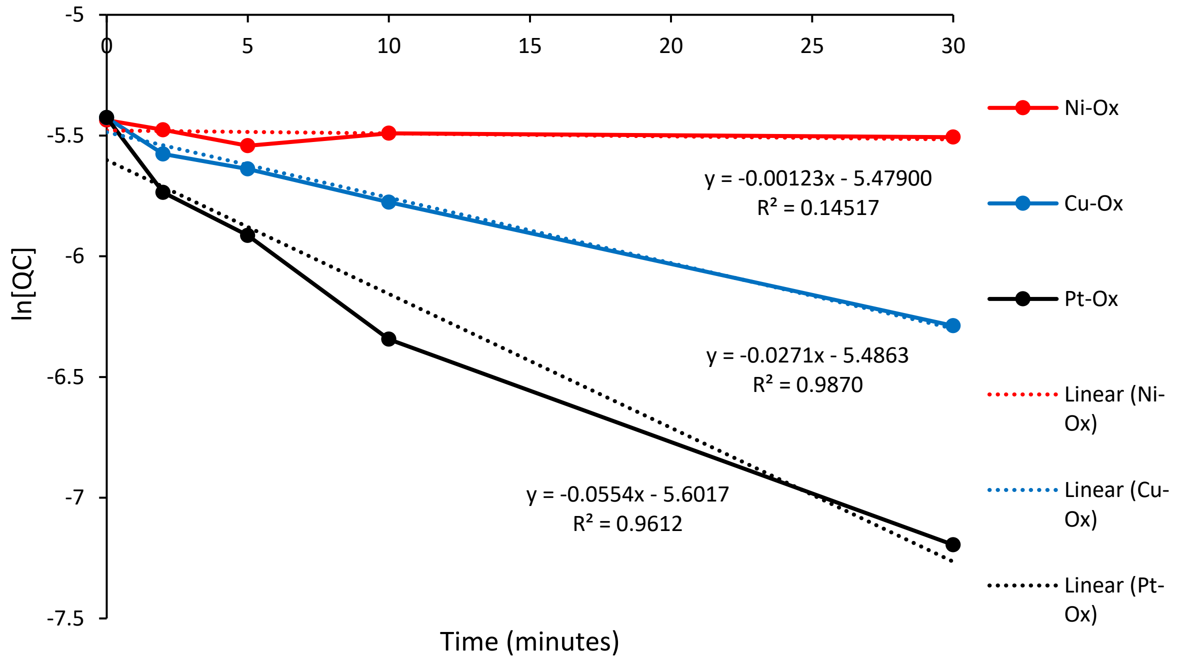

Figure S39 Reaction rates of the catalytic back-reaction from QC to NBD for the oxidized catalysts applying the first order kinetics in the logarithmic form.

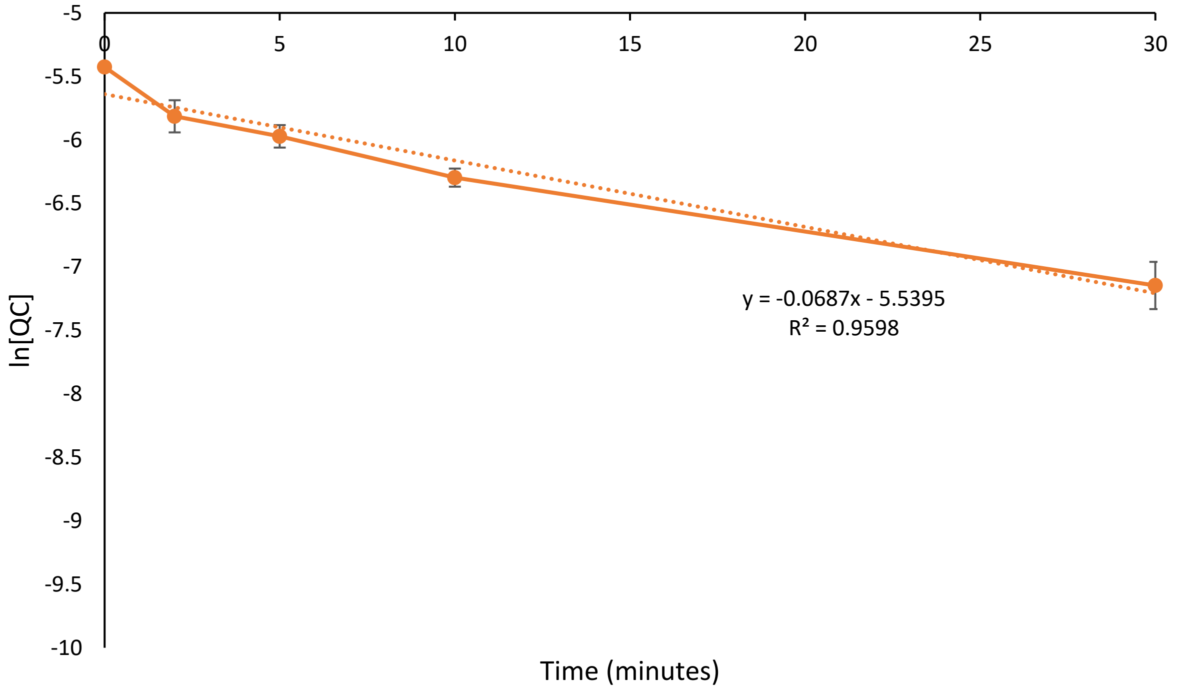

Figure S40 Average of a triplicate of catalytic reactions for the Pt-Ox catalyst applying the first order kinetics in the logarithmic form. Error bars are calculated as the standard deviation between each measurement multiplied by the square root of the total number of measurements.

References

J. Orrego-Hernandez, H. Hölzel, M. Quant, Z. Wang, K. Moth-Poulsen, *Chem. Eur. J.* **2021**, *2021*, 5337-5342.
